# Trends and Inequalities in Overall and Abdominal Obesity by Sociodemographic Factors in Korean Adults, 1998–2018

**DOI:** 10.3390/ijerph18084162

**Published:** 2021-04-14

**Authors:** Ha-Jin Jang, Hannah Oh

**Affiliations:** 1 Interdisciplinary Program in Precision Public Health, Department of Public Health Sciences, Graduate School of Korea University, Seoul 02841, Korea; hajinjang@korea.ac.kr; 2 Division of Health Policy and Management, College of Health Sciences, Korea University, Seoul 02841, Korea

**Keywords:** body fatness, adiposity, obesity, overweight, BMI, body mass index, health disparity

## Abstract

Few studies have comprehensively examined the nationwide trends in overall and abdominal obesity prevalence and related sociodemographic inequalities in Korea. In the Korea National Health and Nutrition Examination Survey 1998–2018, we estimated the age-standardized prevalence of overall (body mass index ≥ 25 kg/m^2^) and abdominal obesity (waist circumference ≥ 90 cm men, ≥85 cm women) in each sociodemographic subgroup of Korean adults (aged 19–79 years). For each survey year, weighted logistic regression was performed to estimate odds ratios (ORs) and 95% confidence intervals (CIs) for the associations between obesity prevalence and sociodemographic factors. During the study period, the prevalence of overall and abdominal obesity increased in men (24.8% to 42.4%; 20.1% to 32.1%; respectively) but only a small change was observed in women (26.5% to 26.0%; 22.7% to 20.9%; respectively). Obesity prevalence increased in all sociodemographic groups of men but varied across groups in women. In women, income (4th vs. 1st quartiles in 2016–2018: OR (95% CI) = 0.66 (0.56–0.78) overall obesity; 0.60 (0.51–0.71) abdominal obesity) and education (college or higher vs. high school or less: 0.62 (0.54–0.72) overall obesity; 0.58 (0.50–0.68) abdominal obesity) were inversely associated with obesity prevalence, and the gaps became more pronounced since 2007. Our data suggest that the inequalities in obesity prevalence by sex and by socioeconomic status have become more apparent over time in Korea.

## 1. Introduction

The prevalence of obesity has increased globally during the past several decades, leading to an era of epidemic obesity. Because obesity (overall as well as abdominal obesity) increases risks of various chronic diseases, including cardiovascular disease [1], type 2 diabetes mellitus [2], and cancer [3], an increase in obesity prevalence has contributed to a rise in disease burden and related medical expenditures in many countries [4,5,6,7].

To date, the increase in obesity prevalence has been most extensively documented in Western countries, including the US and European countries. In these populations, obesity prevalence considerably increased from 1970 to 2000 and stabilized through the early 2010s [8,9,10,11]. However, in other parts of the world, particularly Asian countries with traditionally low obesity prevalence, an increasing trend in obesity prevalence has become apparent only in recent decades [9,12]. South Korea (we refer to “Korea” throughout the manuscript) is one of the Asian countries that has undergone extremely rapid economic growth and Westernization during the past several decades. Since the 1980s, particularly after the 1988 Seoul Olympics, globalization accelerated the introduction of fast-food chains, such as McDonald’s and KFC, and other aspects of a Western-style diet [13,14,15]. With the Westernization of lifestyle, characterized by the high consumption of high-calorie, animal-based foods and low levels of physical activity, the obesity prevalence in Korea is likely to have elevated [16]. To prevent further increase in obesity and related disease burdens in Korea, a comprehensive investigation of nationwide obesity transition and identification of vulnerable populations is required.

Because few studies have examined obesity trends in Korea using more recent data [17,18,19,20,21], little is known about the extent to which the obesity prevalence has changed over time and the time period during which the slope has shifted. Little is also known about the extent to which the obesity prevalence varies by sociodemographic groups and whether the inequalities have changed over time. Previous studies suggested that the obesity prevalence may differ by sociodemographic factors [22,23,24], as these factors may influence the access to knowledge and resources required to practice a healthy lifestyle.

In this study, we examined the nationwide trends and inequalities in overall and abdominal obesity among Korean adults in 1998–2018 using nationally representative, repeated health examination survey data. To further understand the factors that contributed to the inequalities in obesity over time, we investigated the relationships of sociodemographic factors (sex, age, birth cohort, income, education, region, and occupation) with obesity transition. We examined the associations with both overall and abdominal obesity by evaluating changes in mean body mass index (BMI) and waist circumference (WC), as well as obesity prevalence using binary cutpoints.

## 2. Materials and Methods

### 2.1. Study Population

This study included adult participants in the Korea National Health and Nutrition Examination Survey (KNHANES) 1998–2018. The KNHANES is a nationally representative, repeated cross-sectional survey aiming to assess the health and nutritional status of the noninstitutionalized Korean population [25]. The KNHANES was conducted by the Korea Centers for Disease Control and Prevention (KCDC) and the Korea Ministry of Health and Welfare in 1998, 2001, 2005, and annually since 2007. Before 2007, the survey was conducted approximately once every three years (Phase I in 1998, Phase II in 2001, Phase III in 2005), each including about 35,000 participants. Since 2007, the survey has become an ongoing program conducted every year, including about 10,000 participants per year over three-year period (Phase IV: 2007, 2008, 2009; Phase V: 2010, 2011, 2012; Phase VI: 2013, 2014, 2015; Phase VII: 2016, 2017, 2018), to provide timely statistics for monitoring health status of the population. A stratified, multistage clustered probability-based sampling design was used to select the sample units based on sex, age, and geographic area using household registries. The KNHANES consisted of three distinct surveys: the health interview, health examination, and nutrition survey. The health interview and health examination were conducted at mobile examination centers. The health interview collected information on sociodemographic factors, health behaviors, and medical history. Health examination included anthropometric measurements and was performed by trained medical personnel following standardized protocols. All surveys collected comparable data using similar designs and sampling methods. At the time of enrollment, all participants in the survey provided written informed consent. All procedures were approved by the Institutional Review Board of the KCDC.

In this study, we used the KNHANES data from Phase I through Phase VII: Phase I (1998), Phase II (2001), Phase III (2005), Phase IV (2007–2009), Phase V (2010–2012), Phase VI (2013–2015), and Phase VII (2016–2018). Of the 208,705 participants in the KNHANES I to VII, we excluded those who were at age < 19 years (*n* = 52,632) or ≥80 years (*n* = 4242), pregnant (*n* = 563), had missing information on BMI (*n* = 63,074) or WC (*n* = 163), had BMI < 13 or >50 kg/m^2^ (*n* = 2), and had WC < 40 or >140 cm (*n* = 4). There was no significant difference in the distribution of sociodemographic factors between those who were included vs. excluded due to missing information on BMI and WC. After these exclusions, a total of 88,025 participants (38,438 men and 49,587 women) were included in the analysis (participant flow chart in Supplementary Figure S1). The numbers of male and female participants according to survey years and sociodemographic factors are shown in Supplementary Table S1.

### 2.2. Overall and Abdominal Obesity

BMI (the ratio of body weight [kg] to height squared [m^2^]) and WC [1] were used as measures of overall and abdominal obesity, respectively. Body weight, height and WC were measured during health examination by trained medical staff following the standardized protocols in all survey phases. Body weight was measured to the nearest 0.1 kg on a calibrated balance-beam scale wearing a lightweight clothing or underwear without shoes (GL-6000-20; CAS KOREA, Seoul, Korea). Height was measured to the nearest 0.1 cm in the erect position using a stadiometer (SECA 225; SECA, Hamburg, Germany). WC was measured to the nearest 0.1 cm at the midpoint between the lower margin of the rib cage and the top of the iliac crest during minimal respiration (SECA 200, SECA). Based on the Asia-Pacific regional guidelines of the World Health Organization (WHO) and International Obesity Task Force (IOTF) [26], we defined overall obesity as BMI ≥ 25 kg/m^2^. Because the WHO guidelines recommend BMI ≥ 30 kg/m^2^ for non-Asian populations, we also examined the obesity trends using BMI ≥ 30 kg/m^2^ cutpoint to increase comparability of our results with global data. However, in stratified trend and association analyses, we used BMI ≥ 25 kg/m^2^ cutpoint only because the prevalence of BMI ≥ 30 kg/m^2^ was very low in our study population. Abdominal obesity was defined as WC ≥ 90 cm for men and WC ≥ 85 cm for women [27]. In secondary analysis, we also examined the trends in abdominal obesity using waist-to-height ratio (WHtR). Because there is no clear obesity guideline for the WHtR cutpoint, we examined the mean WHtR.

### 2.3. Sociodemographic Variables

Sociodemographic information including age, sex, household income, education, region, and occupation were assessed through face-to-face interview. We categorized age into 10-year intervals: 19–29, 30–39, 40–49, 50–59, 60–69, and 70–79 years. Birth cohort was estimated by subtracting age from the calendar year of survey. Birth cohorts were then categorized into seven groups: 1919–1939, 1940–1949, 1950–1959, 1960–1969, 1970–1979, 1980–1989, and 1990–1999. Income, education, region, and occupation were used as surrogate measures of socioeconomic status (SES) [28,29]. Because SES is a complex trait not adequately represented by a single variable, we used multiple variables that indicate the accessibility to social capitals and healthcare services, as well as other resources and knowledge required to practice healthy lifestyle. For income, equivalized monthly household income ((monthly overall household income)/√(household size)) was calculated to account for the differences in household’s size [30] and categorized into quartiles. Education level was defined as the highest level of education completed by the individual as of the date of the interview. Because the proportion of elementary and middle school graduates was very small, we collapsed the categories into two groups: some high school or less, and some college or higher. Region was categorized into Seoul (capital city), metropolitan urban (Busan, Daegu, Incheon, Gwangju, Daejeon, Ulsan, and Sejong), non-metropolitan urban (dong), and rural areas (eup and myeon). Occupation was categorized into three groups: physical laborer, non-physical laborer, and unemployed.

### 2.4. Statistical Analysis

To account for complex sampling design and adjust the data to represent the entire Korean adult population, weighted analyses were performed using SURVEY procedures (SURVEYFREQ, SURVEYMEANS, SURVEYREG, SURVEYLOGISTIC) in SAS 9.4 (SAS Institute Inc., Cary, NC, USA). We first investigated the temporal trends in overall and abdominal obesity in 1998–2018. To examine the overall trends, we estimated the age-standardized prevalence of overall and abdominal obesity, as well as the age-standardized mean BMI and WC, in each survey phase from the KNHANES I (1998) to VII (2016–2018) using the 2005 Korean Census population as the reference population. We also performed weighted linear regression on obesity prevalence to estimate β coefficients indicating the change in obesity prevalence per 1-unit increment in survey phase, adjusting for age. Tests for trend were performed using the Wald test for continuous survey phase variables. In exploratory analysis, we also performed segmented regression analysis of interrupted time series [31] to detect the time point in which the changes in obesity prevalence and slope of obesity trend occurred. To examine the variation in obesity trends by sociodemographic factors, we estimated the obesity prevalence separately by sociodemographic subgroups. To examine the change in obesity prevalence by age and birth cohort, we estimated the age-specific obesity prevalence within each birth cohort and longitudinally compared the prevalence across the age within the same birth cohorts. Similarly, the difference between birth cohorts was examined within the same age group. We tested for any difference among categories using global F test. To examine the change in mean BMI and WC, all analyses were repeated using BMI and WC as continuous variables.

Next, we investigated the inequalities in overall and abdominal obesity by socioeconomic factors. Within each survey phase, we performed weighted multivariable logistic regression to estimate odds ratios (ORs) and 95% confidence intervals (CIs) for the associations between socioeconomic factors (income, education, region, and occupation) and prevalence of overall and abdominal obesity, simultaneously adjusting for all sociodemographic factors under study. We repeated the analyses using weighted linear regression for continuous BMI and WC variables. Because the obesity trends and associations with sociodemographic factors varied by sex, we conducted all analyses separately by sex. All statistical tests were two-sided, with an alpha level of 0.05.

## 3. Results

### 3.1. Trends of Overall and Abdominal Obesity in 1998–2018

**Figure 1:**
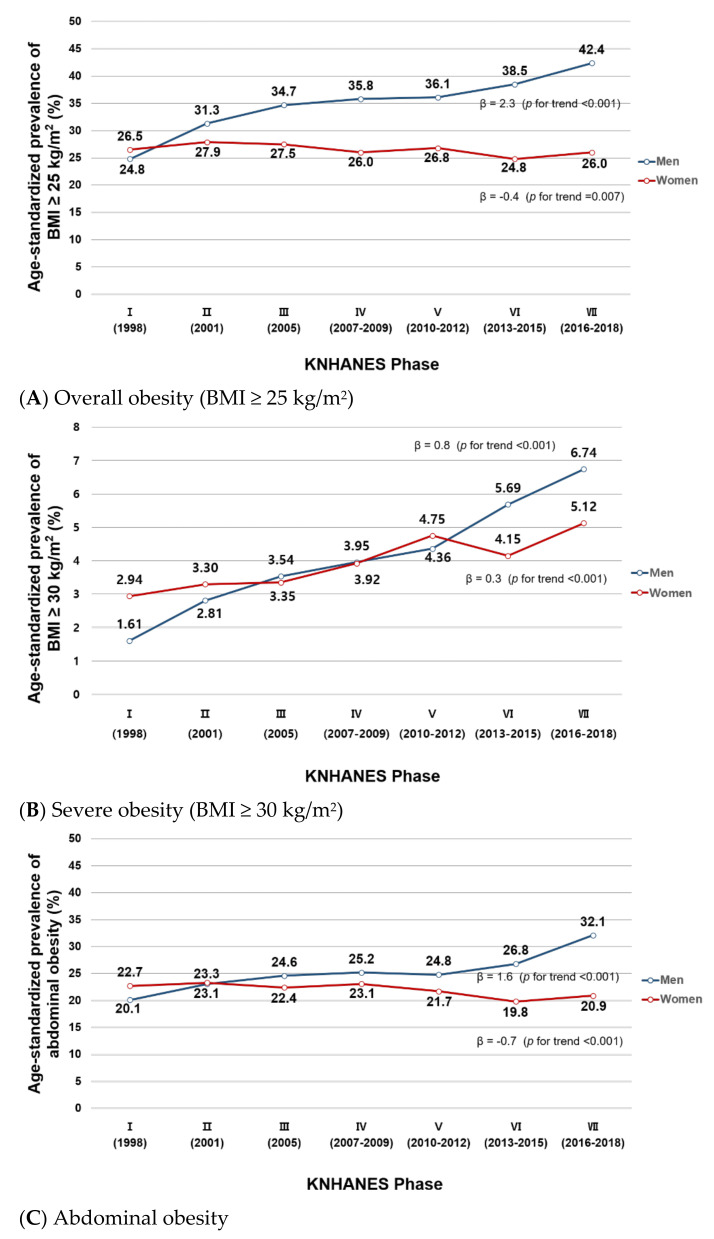
(**A**–**C**). Age-standardized prevalence of overall and abdominal obesity in Korean men and women from KNHANES I (1998) to VII (2016–2018). This figure shows the age-standardized prevalence of (**A**) overall obesity (BMI ≥ 25 kg/m^2^), (**B**) severe obesity (BMI ≥ 30 kg/m^2^), and (**C**) abdominal obesity in Korean men and women in the KNHANES I–VII. Age-standardization was performed using the 2005 Korean Census population as the reference population.

Figure 1 shows the temporal trends in age-standardized prevalence of overall and abdominal obesity in the KNHANES I (1998)–VII (2016–2018). The trends varied by sex. In men, the age-standardized prevalence of overall obesity (BMI ≥ 25 kg/m^2^) statistically significantly increased during the study period, from 24.8% in Phase I to 42.4% in Phase VII (β = 2.3, *p* for trend < 0.001) (Figure 1A). In women, however, there was a minimal change in the age-standardized prevalence of overall obesity (26.5% to 26.0%; β= −0.4, *p* for trend = 0.007). The age-standardized prevalence of severe obesity (BMI ≥ 30 kg/m^2^) increased in both men (1.61% to 6.74%, β = 0.8, *p* for trend < 0.001) and women (2.94% to 5.12%, β = 0.3, *p* for trend < 0.001) (Figure 1B). In abdominal obesity, the age-standardized prevalence increased in men (20.1% in Phase I to 32.1% in Phase VII; β = 1.6, *p* for trend < 0.001) but slightly decreased in women (22.7% to 20.9%; β= −0.7, *p* for trend < 0.001; Figure 1C). In both overall and abdominal obesity, the age-standardized prevalence was similar between men and women in Phase I (overall obesity: 24.8% men vs. 26.5% women; abdominal obesity: 20.1% men vs. 22.7% women) but the gap between the two sexes gradually increased over time (Phase VII: 42.4% men vs. 26.0% women for overall obesity; 32.1% men vs. 20.9% women for abdominal obesity). We observed similar results with trends in mean BMI, WC, and WHtR: increasing trends in men (23.1 to 24.6 kg/m^2^, 82.6 to 86.1 cm, 0.489 to 0.500, respectively) and a minimal change in women (23.1 to 23.1 kg/m^2^, 77.9 to 77.1 cm, 0.499 to 0.486, respectively) during the study period (Supplementary Figure S2).

**Figure 2:**
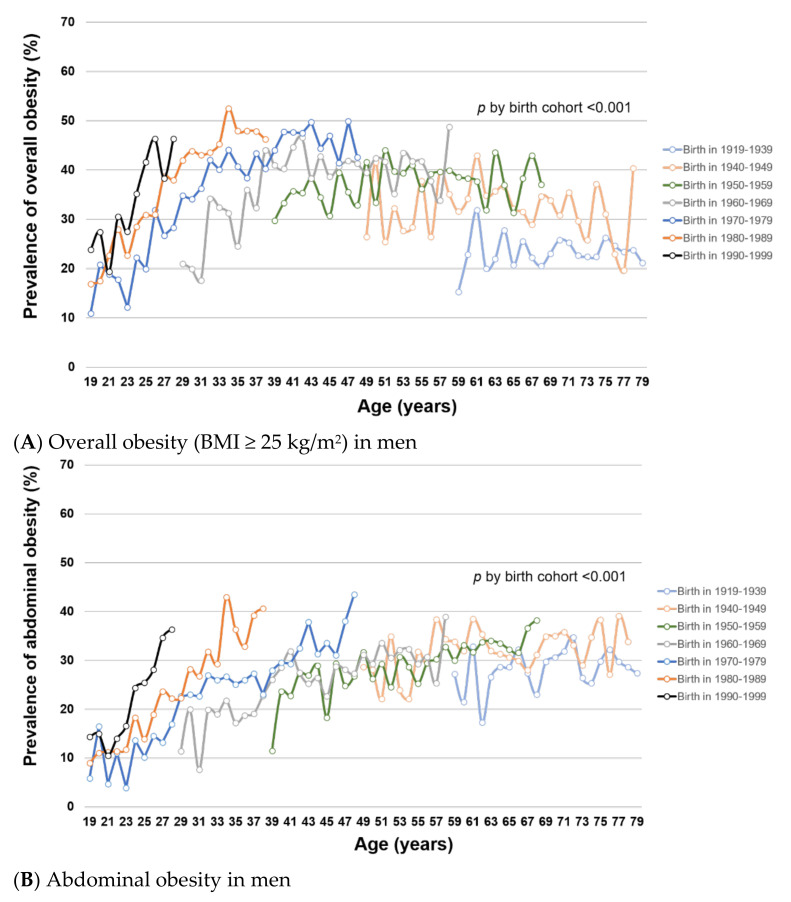

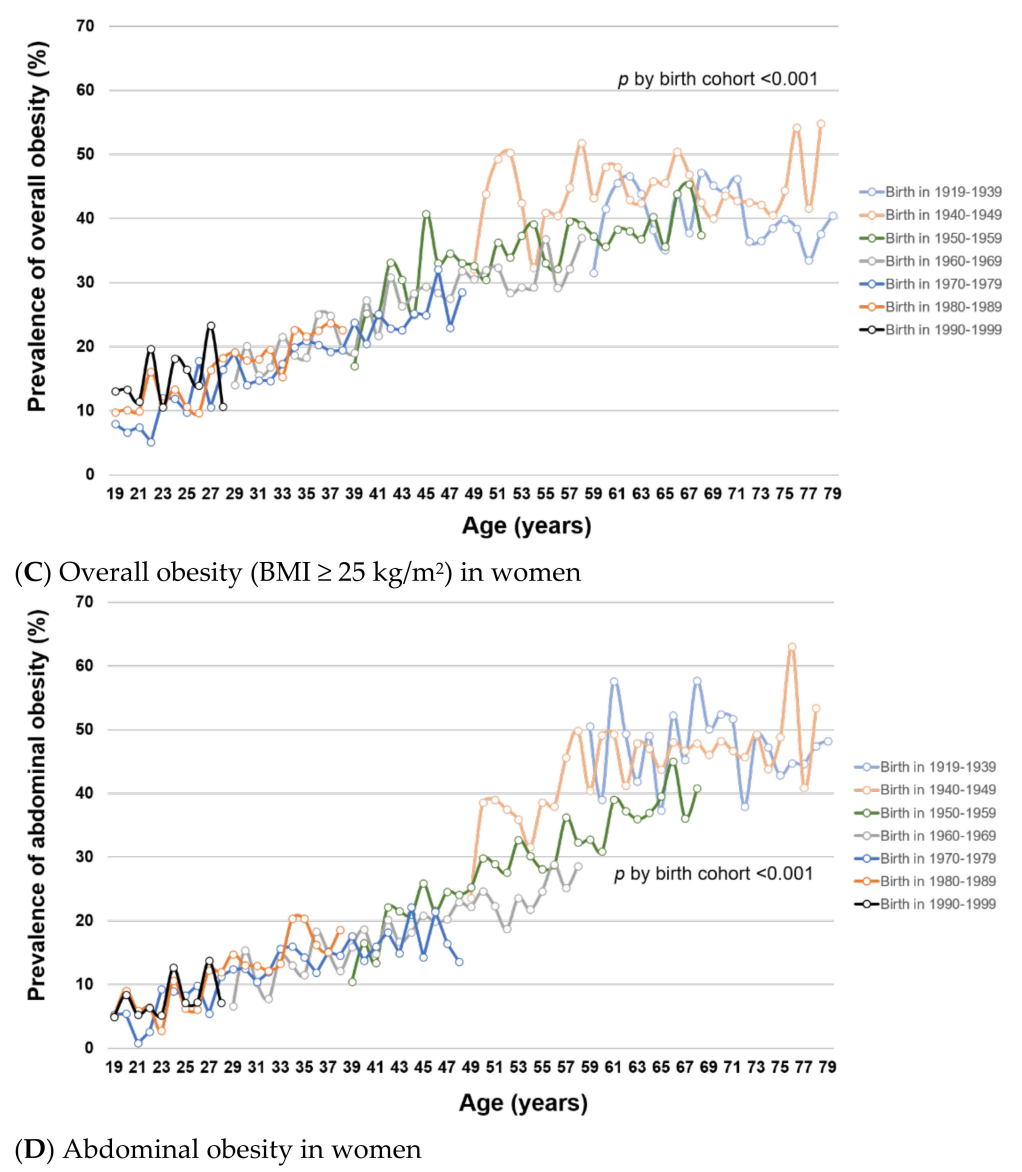
(**A**–**D**). Trends of overall and abdominal obesity prevalence by birth cohort and age in Korean men and women from KNHANES I (1998) to VII (2016–2018). This figure shows the age-specific prevalence of (**A**) overall obesity (BMI ≥ 25 kg/m^2^) in men, (**B**) abdominal obesity in men, (**C**) overall obesity (BMI ≥ 25 kg/m^2^) in women, and (**D**) abdominal obesity in women by birth cohort in KNHANES I–VII. Tests for difference by birth cohorts were performed using global F tests for categorical variable of birth cohorts.

Because the most change in obesity prevalence appeared to occur in Phase VI (Figure 1 and Figure 2), in exploratory analysis we performed a segmented regression analysis comparing prevalence and slope of obesity trend before vs. after the Phase VI. The results are shown in Table S2. For overall obesity, the prevalence and slope of male and female obesity trends did not significantly change in Phase VI. However, for abdominal obesity, the slope of trend in male obesity prevalence significantly increased in Phase VI (β = 0.041, *p* < 0.001).

To examine how obesity prevalence changed with age and birth cohort, we estimated age-specific obesity prevalence within each birth cohort. Within birth cohorts, the prevalence of overall and abdominal obesity generally increased with age in both men (Figure 2A,B, respectively) and women (Figure 2C,D, respectively) (all *p* for trend < 0.001), except for overall obesity among men at age >50 years. For a given age, overall and abdominal obesity prevalence varied by birth cohort (all global F-test *p* < 0.001). In men, the prevalence of overall and abdominal obesity was generally higher in younger birth cohorts than in older birth cohorts. In women, the birth cohorts 1960–1979 had significantly lower prevalence of overall and abdominal obesity compared with older birth cohorts (1919–1959). Among the female birth cohorts 1970–1999, the obesity prevalence was not statistically significantly different. Similar patterns of obesity trends by age and birth cohort were observed using mean BMI and WC (Supplementary Figure S3).

Table 1 presents the trends in prevalence of overall and abdominal obesity from phase I to VII in each subgroup defined by sociodemographic factors. In men, the prevalence of overall and abdominal obesity increased over time in all groups, with age-adjusted β coefficients ranging 1.45–3.18 for overall obesity and 0.23–2.84 for abdominal obesity. In women, the change in obesity prevalence largely varied by sociodemographic groups. The prevalence of overall and abdominal obesity increased over time in women aged 70–79 years and those with some college or higher education (age-adjusted β range: 0.53 to 1.65), while the prevalence decreased in women aged 40–69 years and those with the highest income level (age-adjusted β range: −0.58 to −3.19). Although the prevalence of obesity increased in women with some college or higher education, the prevalence in Phase VII was still higher among women with some high school or lower education (36.5% vs. 18.4% overall obesity, 33.4% vs. 12.5% abdominal obesity). Similar associations with sociodemographic factors were found with mean BMI and WC (Supplementary Table S3).

### 3.2. Assocation between Socioeconoic Factors and Obesity

Table 2 (for men) and Table 3 (for women) present the associations of socioeconomic factors with prevalence of overall and abdominal obesity after mutual adjustment for all sociodemographic factors under study. For men, there was no consistent pattern of association between socioeconomic factors and obesity prevalence during the study period (Table 2). In women, there were significantly inverse associations of income and education with both overall and abdominal obesity prevalence, indicating lower obesity prevalence in higher income and higher education groups (Table 3). The inequalities in female obesity prevalence by income levels were not present in Phase I but became pronounced since Phase IV. Women with higher income levels had significantly lower prevalence of overall and abdominal obesity in the latest 4 surveys (KNHANES IV–VII), with OR ranging 0.61–0.84 for overall obesity and 0.54–0.83 for abdominal obesity. The inequalities in obesity prevalence by education levels (some college or higher vs. some high school or less) were present from Phase I and persisted throughout the entire study period, with OR ranging 0.43–0.62 for overall obesity and 0.39–0.69 for abdominal obesity. In several survey years, women living in rural and non-metro urban areas (vs. Seoul) and women who were unemployed and in physical labor (vs. non-physical labor) had higher obesity prevalence. To further examine the difference in obesity prevalence between rural vs. urban areas, we compared the prevalence within the similar occupation types. Among women who were unemployed or in physical labor, the obesity prevalence was consistently higher in women living in rural and non-metro urban areas (vs. Seoul and metro-urban areas) and the associations were statistically significant in Phase V (OR (95% CI) = 1.20 (1.05, 1.37) overall obesity; 1.18 (1.02, 1.36) abdominal obesity) and Phase VI (1.26 (1.12, 1.42); 1.31 (1.13, 1.51); respectively) (Supplementary Table S4). Among women in non-physical labor, similar obesity prevalence was observed by region. Similar patterns of associations with socioeconomic factors were observed using mean BMI and WC (Supplementary Tables S5 and S6).

## 4. Discussion

Using nationally representative, repeated survey data from 1998 to 2018, this study comprehensively examined the nationwide obesity transition and its associations with sociodemographic factors in Korean men and women. In men, the prevalence of overall and abdominal obesity increased in all sociodemographic subgroups. Throughout the study period, the obesity prevalence in men was similar among the subgroups, indicating little inequality in Korean men. However, in women, the obesity trends varied among sociodemographic subgroups. Decreasing trends in obesity prevalence were observed in certain age groups (40–69 years) of women and in women with high income levels, while increasing trends were observed in others. Further, women with higher income and education levels had lower obesity prevalence and the gap between the groups became more pronounced over time, indicating an increasing inequality in obesity by SES in Korean women. Because obesity is an important risk factor for many chronic diseases, our data suggest that the inequalities in the burden of diseases are also likely to increase in the future, particularly among women.

While most studies have focused on the cross-sectional differences in obesity prevalence among different subgroups [22,23,24,32], our study examined the temporal trends in obesity inequalities. During the 20-year period (1998–2018), we observed an obesity transition in Korea as summarized in Supplementary Figure S4 (4A for overall obesity and 4B for abdominal obesity), a shift from the “low prevalence, little inequality” stage to the “higher prevalence, greater inequality” stage. Korea has undergone a remarkable change in its economy and lifestyle during the study period. Immediately after the Korean War in 1950–1953, the food insecurity and undernutrition were important public health problems in Korea. However, with rapid industrialization and globalization, various fast-food chains and western-style diet have become widely available particularly since the 1990s. Our findings suggest that such changes may have unequally influenced the populations of different sex, age, birth cohort, and SES, leading to the increased inequalities over time. The inequality between high vs. low SES are also observed in other Organization for Economic Cooperation and Development (OECD) member countries with higher obesity prevalence such as the US [32]. The access to resources and knowledge may influence individual’s ability to maintain healthy weight [33,34]. Today, healthy food options and lifestyles are often more costly options [35]. Further, obese individuals are at higher risk of chronic diseases [36,37], social stigma [38,39,40,41], and depression [41,42,43,44,45]. The vicious cycle of obesity may negatively influence the job accessibility and economic status in the obese individuals [46,47,48], strengthening the relationship between SES and obesity. As we observed in the present study, many OECD member countries have also reported greater socioeconomic inequalities in obesity among women than among men [32]. The difference may be partly explained by stronger relationships of obesity with unemployment and social stigma in women (vs. men) [46,49]. Among the populations of lower SES, men and women may also have different occupation types and lifestyle patterns. The commonly held occupations in lower SES are the high activity, physical labor jobs among men but unemployment and service jobs among women. In our study population, we observed that 67% of lower SES men were physical laborers, whereas 48% and 20% of lower SES women were unemployed and service workers, respectively, in Phase VII. Among unemployed/physical labor women, the difference in obesity prevalence was also observed between rural/non-metro vs. Seoul/metro urban areas. The higher obesity prevalence in rural/non-metro areas may be partly due to the remaining differences in SES and the limited accessibility to diverse food sources, sports facilities, and healthcare services in rural areas. The rural/urban inequalities are also observed in many other countries [50,51,52] and thus more research is needed to understand the role of region (rural vs. urban) in obesity transition. Further, the different inequality patterns by SES in men vs. women may indicate that the factors contributing to obesity development may also differ by sex.

In the present study, we also identified the sex inequalities in obesity among Korean adults. During the study period, the overall and abdominal obesity prevalence increased by about 70% and 60%, respectively, in men. The changes we observed over the 20-year period is likely to be clinically significant. However, in women only a small change was observed, leading to an increased gap between the two sexes over time. In 1998, both overall and abdominal obesity prevalence were about 2% higher in women compared with men but the pattern rapidly reversed, with the prevalence in men consistently being higher since 2005. In 2016–2018, the difference in obesity prevalence between men and women increased to 16.4% for overall obesity and 11.2% for abdominal obesity. The discrepancy in obesity trends between men and women may be partly explained by the differences in trends of total energy intake and energy expenditure. In Korean men, the total energy intake increased from 2196 to 2489 kcal/d in the KNHANES I–VI [53], while the percentage of men achieving the recommended level of physical activity decreased [54]. The increase in total energy intake in men was primarily driven by an increased consumption of sugar-sweetened beverages [53] and an increased frequency of eating out [55]. In Korean women, the percentage of women achieving the recommended level of physical activity decreased, but there was no change in total energy intake (1753 to 1754 kcal/d). The decrease in activity levels among Korean men and women may be partly due to the shift in occupational structure (e.g., shift from physical labor to non-physical, clerical jobs), as well as the changes in lifestyle. The proportion of population participating in physical labor jobs decreased during the study period (58% to 41% in men, 38% to 30% in women), while the proportion of population participating in non-physical labor jobs increased (19% to 29% in men, 10% to 22% in women) (Supplementary Table S1). In women, the proportion of unemployed population has also decreased, possibly mitigating the effects of decreasing activity levels. Other factors including social pressure for thin body shape and fertility rate reduction may also have influenced the obesity prevalence in women. In the present study, we also observed that the male obesity prevalence was particularly higher in younger (vs. older) birth cohorts. Younger birth cohorts are more likely to have exposed to the obesogenic lifestyles such as high-calorie diet, prolonged sedentary time, and inadequate exercise from the early life. The exposure to the obesogenic lifestyles from the early childhood and adolescence may help establish these behaviors through the adulthood, increasing the lifelong risk of obesity. These findings suggest that the past and current obesity prevention policies and interventions have not been successful in men, particularly among younger generations of men [56]. On the contrary, it is possible that the yearning for a slimmer body shape and losing weight [57,58] may play a role in counteracting the effects of lifestyle change in young Korean women. Further studies are needed to better understand the differential effects of birth cohort in men vs. women. A formal age-period-cohort analysis may also help clarify the role of birth cohort effects in obesity transition among Korean adults.

Our observation of lower obesity prevalence in women (vs. men) is a unique feature of obesity transition in Korea compared with those observed in other countries at similar stages of economic development [59]. Particularly in East Asian countries such as Korea, women experience strong stigma and social pressure demanding for thinner body shapes [60,61,62]. The discrepancy between the perceived body image and the actual weight is also stronger in Korean women compared with Korean men, as well as compared with women from other countries [63,64,65]. Furthermore, the increased exposure to social media as well as the celebrities (e.g., K-Pop stars) with slender body shapes in Korea may exacerbate the yearning for and pressure of being thin in young women [66].

In the present study, we also observed that, within birth cohorts, the obesity prevalence increased with age, especially in women. The biological processes of ageing [67,68,69,70,71] include decreased metabolism, reduced muscle mass, and a concomitant increase in whole-body fat mass [71,72]. In women, the hormonal fluctuation and the changes in body fat distribution also occur during the peri- and post-menopausal period. At menopause, ovaries stop producing estrogens, a key hormone that involves in regulation of metabolism, lipolysis/lipogenesis, locomotor activity and energy expenditure [69]. The shift in the ratio of reproductive steroids such as estrogen and adrenal androgen can also increase the risk of abdominal obesity and visceral fat accumulation in lower body parts [73,74,75].

The present study has several limitations. First, we defined overall obesity based on BMI, which is calculated using body weight and height. While obesity indicates excessive body fat, BMI does not distinguish body fat from muscle mass and thus may lead to an inaccurate estimation of obesity prevalence in the population. Particularly, in elderly population, BMI may be a poor proxy for adiposity. However, in the present study, we observed similar trends and associations using WC and WHtR, suggesting that our findings with BMI-based measures are likely to represent those with adiposity. Second, we defined overall obesity as BMI ≥ 25 kg/m^2^ following the WHO guidelines for Asia-Pacific population. Therefore, our results should be interpreted with caution when comparing with findings from other populations because our results also include participants who would have been classified as overweight using other guidelines commonly used in Western countries (BMI ≥ 30 kg/m^2^). Third, many subjects (*n* = 63,243) were excluded due to missing anthropometric measurements, primarily from the first three surveys (Phase I-III). We cannot exclude the possibility of selection bias if the missingness occurred differentially in obese vs. non-obese populations. However, we confirmed that the distributions of sociodemographic factors were similar between those who were included vs. excluded from our analysis. Fourth, we used income, education, region, and occupation as surrogate measures of SES. It is possible that our study did not fully capture the complex trait of SES, such as subjective social class and relative poverty. Lastly, we were not able to examine the obesity prevalence and trends prior to 1998 because the nationwide obesity surveillance such as the KNHANES was not available prior to 1998. Pilot national nutrition surveys conducted in 1969–1989 included a small number of households and were restricted to specific regions and age groups. Based on these earlier surveys, it is likely that the obesity prevalence in Korean adults used to be very low due to the insufficient nutritional intake and the prevalence started to increase after the 1970s [14,76], as the food shortage and undernutrition were highly prevalent until the 1970s [76].

The present study also has important strengths. First, we used nationally representative data applying sampling weights in all analyses. Second, our study included the entire survey phases of the KNHANES with the most recent survey from 2018. Therefore, this is the largest and the most recent study to investigate the secular trends and inequalities in obesity prevalence among Korean adults, allowing most comprehensive examination of nationwide obesity transition. By including more recent data through 2018, we were able to identify additional findings. While earlier studies of KNHANES [18,21] suggested that the male obesity prevalence reached a plateau since 2010, our study observed a further increase in the prevalence in 2013–2018. Our segmented analysis further supported that the male abdominal obesity prevalence has not leveled off, showing a steeper increasing slope in 2013–2018. Second, the anthropometric data were collected by direct measurement rather than self-report and thus the information is expected to be more accurate. We also evaluated both overall and abdominal obesity as these factors independently influence disease risk. Lastly, we stratified the analyses of obesity trends by various demographic and socioeconomic factors and identified the contribution of these factors to obesity inequalities in Korea. In these analyses, we observed distinct patterns of obesity transition in different sociodemographic groups, providing evidence of the need for targeted public health plans and customized obesity prevention strategies.

## 5. Conclusions

In conclusion, the prevalence of overall and abdominal obesity increased over time in Korean men but not in Korean women from 1998 to 2018. While in men, the obesity prevalence increased in all groups, in women, the prevalence decreased in groups with high income levels. The inequalities in obesity prevalence between men vs. women and between women at low vs. high socioeconomic levels have also become more apparent in recent years. Our data suggest that targeted, customized preventive strategies (e.g., precision nutrition policy), particularly in the susceptible populations, are needed to prevent further increases in obesity and related disease burdens in Korea. Further studies are also needed to investigate the related mechanisms and preventive strategies that reduce the SES and sex inequalities in obesity prevalence.

## Figures and Tables

**Table 1:** Prevalence of overall and abdominal obesity by sociodemographic factors in Korean men and women: the KNHANES I–VII.

Prevalence of Overall Obesity (%)																			
		Men									Women								
Sociodemographic Characteristics		I (1998)	II (2001)	III (2005)	IV (07–09)	V (10–12)	VI (13–15)	VII (16–18)	Age-Adjusted β	*p* for Trend	I (1998)	II (2001)	III (2005)	IV (07–09)	V (10–12)	VI (13–15)	VII (16–18)	Age-Adjusted β	*p* for Trend
**Age**	**19–29 years**	18.5	24.8	24.8	30.3	28.3	31.3	38.0	2.65	<0.001	11.7	11.5	13.4	13.6	14.2	14.0	15.8	0.64	0.01
	**30–39 years**	27.9	34.4	38.1	39.4	41.2	44.9	47.8	2.94	<0.001	20.4	19.6	19.0	16.5	21.5	18.8	21.0	0.26	0.29
	**40–49 years**	33.5	38.7	41.1	40.3	42.9	42.3	47.1	1.75	<0.001	29.9	34.1	29.0	27.2	29.2	24.5	26.7	−0.97	0.001
	**50–59 years**	27.6	32.2	41.0	41.6	34.8	40.8	41.6	1.45	<0.001	42.5	40.6	43.1	38.2	35.1	33.1	31.2	−2.17	<0.001
	**60–69 years**	19.9	26.3	31.0	32.2	35.2	35.0	38.1	2.38	<0.001	38.8	46.2	47.1	47.0	43.1	40.3	38.3	−1.31	<0.001
	**70–79 years**	8.3	23.7	27.1	21.1	26.1	29.0	31.0	2.64	<0.001	34.7	36.3	37.3	39.8	37.6	42.1	44.5	1.65	<0.001
**Income**	**1st Quartile**	22.2	31.7	33.7	35.1	32.0	37.0	40.9	2.32	<0.001	25.5	32.0	29.6	30.5	33.0	31.9	33.5	0.45	0.09
	**2nd Quartile**	24.8	29.5	32.6	35.9	38.2	40.6	41.4	2.59	<0.001	27.9	28.6	28.4	29.8	29.2	27.7	29.6	−0.52	0.05
	**3rd Quartile**	24.0	33.5	35.6	38.3	36.3	38.0	43.5	2.29	<0.001	24.6	26.1	30.4	24.6	27.4	27.1	27.1	−0.39	0.13
	**4th Quartile**	28.4	30.8	37.7	37.1	38.4	38.8	42.6	1.91	<0.001	24.1	24.1	23.8	24.2	23.3	21.5	22.4	−1.11	<0.001
**Education**	**High school or less**	25.1	31.7	35.6	36.1	35.1	37.5	40.1	2.19	<0.001	29.1	33.7	34.6	34.1	35.5	34.0	36.5	−0.07	0.68
	**College or higher**	24.8	31.0	33.9	36.8	37.7	38.7	43.6	2.25	<0.001	11.5	9.7	12.7	13.2	15.9	16.0	18.4	1.00	<0.001
**Region**	**Seoul**	23.5	29.8	32.0	37.8	36.8	37.7	40.8	2.20	<0.001	25.3	24.9	24.1	25.1	23.8	23.1	25.7	−0.80	0.003
	**Metro urban**	27.9	30.9	34.2	34.3	35.2	37.3	39.4	1.69	<0.001	23.3	24.4	28.2	28.2	27.3	26.1	27.7	−0.36	0.15
	**Non-metro urban**	25.7	34.0	35.9	37.6	37.7	39.9	43.3	2.23	<0.001	24.1	27.1	26.2	27.1	27.8	27.0	27.4	−0.47	0.04
	**Rural**	21.9	29.5	37.9	35.0	34.2	38.9	45.5	3.18	<0.001	30.5	35.0	36.0	29.1	36.3	34.3	35.6	0.47	0.17
**Occupation**	**Non-physical labor**	27.8	36.2	38.6	41.0	42.2	40.3	45.6	1.78	<0.001	13.5	9.8	13.5	14.3	17.7	17.6	16.8	0.31	0.28
	**Physical labor**	26.5	32.7	37.0	36.2	35.5	38.6	42.2	2.04	<0.001	31.3	34.5	32.5	31.5	32.7	30.5	32.6	−0.63	0.01
	**Unemployed**	19.4	24.4	27.1	31.2	30.6	34.2	36.8	2.57	<0.001	24.6	27.5	29.5	28.8	29.5	28.0	31.3	−0.04	0.82
**Prevalence of Abdominal Obesity (%)**																			
**Age**	**19–29**	12.3	11.2	11.4	17.6	15.2	17.7	23.6	1.89	<0.001	7.5	6.8	8.0	8.5	8.7	8.8	8.1	0.18	0.36
	**30–39**	16.0	23.1	21.6	23.1	26.7	28.6	35.4	2.84	<0.001	12.9	13.7	12.9	13.3	14.9	14.9	16.0	0.55	0.02
	**40–49**	27.3	28.6	26.7	27.6	26.5	29.3	34.3	1.15	<0.001	22.8	21.5	19.0	20.1	19.4	16.1	19.9	−0.58	0.03
	**50–59**	28.4	27.2	37.5	30.7	27.0	29.3	32.1	0.23	0.51	38.6	37.7	37.6	36.2	29.7	23.7	24.5	−3.19	<0.001
	**60–69**	24.0	26.9	32.7	32.5	31.6	29.6	35.7	1.23	<0.05	44.9	50.2	47.4	50.5	45.1	38.6	38.0	−2.38	<0.001
	**70–79**	13.7	29.8	32.9	28.2	29.5	31.9	36.4	2.27	<0.001	40.7	45.8	44.8	47.1	44.4	47.7	49.9	1.13	0.02
**Income**	**1st Quartile**	17.1	21.8	22.8	24.4	22.9	27.1	32.4	2.04	<0.001	23.1	26.7	26.5	28.8	28.0	28.3	29.1	0.04	0.88
	**2nd Quartile**	19.0	18.6	24.5	25.8	25.4	27.6	32.0	1.85	<0.001	23.2	23.3	21.1	26.5	24.6	21.4	26.0	−0.67	<0.05
	**3rd Quartile**	18.4	23.3	24.7	26.4	24.5	26.9	32.9	1.60	<0.001	19.8	21.7	26.2	20.8	22.6	20.9	21.2	−0.94	<0.001
	**4th Quartile**	22.6	23.4	25.2	25.7	26.9	26.8	31.9	1.07	<0.001	20.4	19.8	18.8	21.9	18.8	17.5	18.2	−1.35	<0.001
**Education**	**High school or** **less**	20.1	22.7	28.3	27.0	26.3	28.8	33.2	1.39	<0.001	25.0	28.4	29.8	32.1	31.4	29.4	33.4	−0.54	<0.05
	**College or higher**	17.9	21.5	19.4	23.9	23.8	25.9	31.9	1.81	<0.001	8.2	7.0	6.8	9.4	10.3	10.7	12.5	0.53	<0.05
**Region**	**Seoul**	19.5	23.7	23.1	24.6	22.2	28.8	33.0	1.65	<0.001	21.7	21.3	18.6	21.9	16.7	16.3	22.6	−1.08	<0.001
	**Metro urban**	20.2	19.9	26.5	26.1	27.6	26.4	29.6	1.06	<0.001	18.7	21.1	25.2	25.5	24.3	21.9	21.6	−1.05	<0.001
	**Non-metro urban**	19.6	23.2	22.6	24.9	23.2	26.6	32.7	1.70	<0.001	20.0	19.3	21.3	22.9	22.6	22.3	23.3	−0.46	0.05
	**Rural**	17.9	22.2	26.0	26.6	27.9	27.3	35.8	2.20	<0.001	27.4	32.8	28.4	30.5	32.9	29.3	31.2	−0.07	0.82
**Occupation**	**Non-physical labor**	18.8	24.4	22.0	27.2	26.6	28.6	35.4	2.07	<0.001	7.8	4.9	7.9	10.0	12.2	11.3	11.1	0.14	0.53
	**Physical labor**	21.0	23.3	26.2	25.0	24.7	26.5	31.4	1.18	<0.001	25.5	26.3	27.0	27.8	27.1	23.4	26.6	−0.95	<0.001
	**Unemployed**	16.2	18.0	23.0	24.9	24.0	27.0	30.2	1.72	<0.001	22.1	24.9	25.0	27.3	25.8	25.2	28.8	−0.34	0.06

Note: Overall obesity was defined as BMI ≥ 25 kg/m^2^. Abdominal obesity was defined as waist circumference ≥90 cm in men and ≥85 cm in women. Values for obesity prevalence are not adjusted for age. β coefficients indicate the change in obesity prevalence per 1-unit increment in survey phase, adjusting for age. β coefficients were estimated using weighted linear regression models on obesity prevalence, adjusting for age (19–29, 30–39, 40–49, 50–59, 60–69, 70–79 years) as a continuous variable. Tests for trend were performed using Wald test for continuous survey phase variable.

**Table 2:** Odds ratios (OR) and 95% confidence intervals (CI) for the associations of socioeconomic factors (income, education, region, occupation) with overall and abdominal obesity prevalence in men: the KNHANES I–VII.

Overall Obesity															
Socioeconomic Characteristics		I (1998)		II (2001)		III (2005)		IV (2007–2009)		V (2010–2012)		VI (2013–2015)		VII (2016–2018)	
		OR	(95% CI)	OR	(95% CI)	OR	(95% CI)	OR	(95% CI)	OR	(95% CI)	OR	(95% CI)	OR	(95% CI)
**Income**	**1st Quartile**	1.00		1.00		1.00		1.00		1.00		1.00		1.00	
	**2nd Quartile**	1.11	(0.85 1.44)	0.88	(0.68, 1.14)	0.96	(0.70, 1.31)	1.03	(0.86, 1.22)	1.24	(1.03, 1.50)	1.17	(0.98, 1.40)	0.99	(0.84, 1.17)
	**3rd Quartile**	1.07	(0.80, 1.44)	1.00	(0.77, 1.30)	1.07	(0.78, 1.48)	1.12	(0.94, 1.34)	1.12	(0.92, 1.36)	0.98	(0.83, 1.17)	1.08	(0.93, 1.26)
	**4th Quartile**	1.29	(0.97, 1.71)	0.89	(0.67, 1.17)	1.21	(0.88, 1.67)	1.05	(0.87, 1.27)	1.23	(1.02, 1.49)	1.06	(0.90, 1.26)	1.04	(0.89, 1.22)
**Education**	**High school or less**	1.00		1.00		1.00		1.00		1.00		1.00		1.00	
	**College or higher**	1.04	(0.84, 1.28)	1.02	(0.82, 1.28)	1.04	(0.78, 1.38)	0.98	(0.85, 1.14)	1.04	(0.89, 1.22)	1.06	(0.92, 1.22)	1.05	(0.92, 1.20)
**Region**	**Seoul**	1.00		1.00		1.00		1.00		1.00		1.00		1.00	
	**Metro urban**	1.25	(0.96, 1.63)	1.03	(0.76, 1.39)	1.07	(0.74, 1.55)	0.86	(0.71, 1.04)	0.93	(0.78, 1.10)	0.97	(0.81, 1.15)	0.94	(0.80, 1.10)
	**Non-metro urban**	1.10	(0.82, 1.47)	1.16	(0.88, 1.53)	1.12	(0.78, 1.61)	0.95	(0.80, 1.12)	1.05	(0.89, 1.24)	1.08	(0.92, 1.26)	1.10	(0.95, 1.26)
	**Rural**	0.99	(0.75, 1.32)	0.93	(0.69, 1.26)	1.25	(0.86, 1.84)	0.90	(0.75, 1.10)	0.97	(0.80, 1.19)	1.03	(0.84, 1.26)	1.27	(1.02, 1.60)
**Occupation**	**Non-physical labor**	1.00		1.00		1.00		1.00		1.00		1.00		1.00	
	**Physical labor**	1.02	(0.81, 1.29)	0.87	(0.68, 1.12)	1.03	(0.78, 1.35)	0.87	(0.73, 1.03)	0.82	(0.70, 0.96)	1.01	(0.87, 1.18)	0.94	(0.82, 1.08)
	**Unemployed**	0.92	(0.67, 1.27)	0.71	(0.50, 1.01)	0.85	(0.59, 1.22)	0.86	(0.70, 1.06)	0.81	(0.65, 0.99)	1.06	(0.87, 1.30)	0.88	(0.74, 1.06)
**Abdominal Obesity**															
**Income**	**1st Quartile**	1.00		1.00		1.00		1.00		1.00		1.00		1.00	
	**2nd Quartile**	1.13	(0.86, 1.48)	0.82	(0.60, 1.13)	1.15	(0.84, 1.59)	1.07	(0.88, 1.29)	1.12	(0.92, 1.37)	1.02	(0.85, 1.23)	0.93	(0.79, 1.10)
	**3rd Quartile**	1.11	(0.84, 1.46)	1.01	(0.76, 1.34)	1.14	(0.78, 1.64)	1.10	(0.90, 1.35)	1.06	(0.86, 1.30)	0.93	(0.78, 1.11)	0.98	(0.83, 1.14)
	**4th Quartile**	1.30	(1.00, 1.68)	1.00	(0.72, 1.37)	1.28	(0.91, 1.79)	1.03	(0.84, 1.26)	1.22	(0.98, 1.51)	0.95	(0.79, 1.13)	0.91	(0.76, 1.08)
**Education**	**High school or less**	1.00		1.00		1.00		1.00		1.00		1.00		1.00	
	**College or higher**	1.18	(0.92, 1.51)	1.22	(0.97, 1.55)	0.95	(0.67, 1.35)	1.06	(0.89, 1.26)	1.02	(0.85, 1.22)	1.00	(0.85, 1.16)	0.99	(0.85, 1.16)
**Region**	**Seoul**	1.00		1.00		1.00		1.00		1.00		1.00		1.00	
	**Metro urban**	1.04	(0.78, 1.39)	0.76	(0.55, 1.05)	1.18	(0.84, 1.64)	1.04	(0.84, 1.30)	1.34	(1.09, 1.65)	0.88	(0.73, 1.06)	0.86	(0.72, 1.03)
	**Non-metro urban**	1.00	(0.71, 1.39)	0.90	(0.67, 1.23)	0.93	(0.68, 1.28)	0.95	(0.76, 1.19)	1.11	(0.90, 1.38)	0.95	(0.80, 1.13)	1.00	(0.85, 1.17)
	**Rural**	0.86	(0.62, 1.20)	0.78	(0.54, 1.13)	0.97	(0.68, 1.39)	1.03	(0.81, 1.31)	1.37	(1.08, 1.74)	0.93	(0.75, 1.16)	1.14	(0.91, 1.42)
**Occupation**	**Non-physical labor**	1.00		1.00		1.00		1.00		1.00		1.00		1.00	
	**Physical labor**	1.18	(0.90, 1.54)	0.99	(0.74, 1.33)	1.13	(0.79, 1.63)	0.85	(0.70, 1.03)	0.86	(0.72, 1.04)	0.88	(0.74, 1.04)	0.81	(0.69, 0.95)
	**Unemployed**	1.10	(0.79, 1.52)	0.92	(0.63, 1.36)	1.13	(0.74, 1.72)	0.88	(0.70, 1.09)	0.96	(0.76, 1.22)	1.05	(0.84, 1.30)	0.83	(0.69, 1.01)

Note: Overall obesity was defined as BMI ≥ 25 kg/m^2^. Abdominal obesity was defined as waist circumference ≥90 cm in men. Mutually adjusted for age (19–29, 30–39, 40–49, 50–59, 60–69, 70–79 years), income level (1st, 2nd, 3rd, 4th quartiles), education level (some high school or less, some college or higher), region (Seoul, metro urban, non-metro urban, rural) and occupation (non-physical labor, physical labor, unemployed).

**Table 3:** Odds ratios (OR) and 95% confidence intervals (CI) for the associations of socioeconomic factors (income, education, region, occupation) with overall and abdominal obesity prevalence in women: the KNHANES I–VII.

Overall Obesity															
Socioeconomic Characteristics		I (1998)		II (2001)		III (2005)		IV (2007–2009)		V (2010–2012)		VI (2013–2015)		VII (2016–2018)	
		OR	(95% CI)	OR	(95% CI)	OR	(95% CI)	OR	(95% CI)	OR	(95% CI)	OR	(95% CI)	OR	(95% CI)
**Income**	**1st Quartile**	1.00		1.00		1.00		1.00		1.00		1.00		1.00	
	**2nd Quartile**	1.18	(0.96, 1.45)	0.88	(0.68, 1.14)	1.00	(0.76, 1.32)	0.94	(0.79, 1.12)	0.87	(0.74, 1.01)	0.82	(0.71, 0.96)	0.84	(0.73, 0.98)
	**3rd Quartile**	1.00	(0.80, 1.25)	0.80	(0.62, 1.04)	1.16	(0.90, 1.49)	0.77	(0.65, 0.91)	0.84	(0.71, 0.99)	0.80	(0.68, 0.93)	0.80	(0.68, 0.93)
	**4th Quartile**	0.99	(0.78, 1.26)	0.86	(0.65, 1.14)	0.86	(0.64, 1.16)	0.76	(0.64, 0.91)	0.71	(0.60, 0.85)	0.61	(0.52, 0.73)	0.66	(0.56, 0.78)
**Education**	**High school or less**	1.00		1.00		1.00		1.00		1.00		1.00		1.00	
	**College or higher**	0.58	(0.44, 0.78)	0.43	(0.32, 0.59)	0.49	(0.36, 0.67)	0.55	(0.45, 0.67)	0.54	(0.46, 0.64)	0.62	(0.53, 0.73)	0.62	(0.54, 0.72)
**Region**	**Seoul**	1.00		1.00		1.00		1.00		1.00		1.00		1.00	
	**Metro urban**	0.89	(0.69, 1.14)	0.82	(0.64, 1.06)	1.10	(0.83, 1.47)	1.09	(0.91, 1.31)	1.17	(0.96, 1.42)	1.07	(0.89, 1.28)	0.98	(0.84, 1.15)
	**Non-metro urban**	0.90	(0.73, 1.11)	1.02	(0.79, 1.31)	1.06	(0.78, 1.43)	1.15	(0.96, 1.38)	1.18	(0.99, 1.39)	1.20	(1.01, 1.42)	1.00	(0.86, 1.16)
	**Rural**	0.90	(0.70, 1.15)	1.00	(0.76, 1.32)	1.27	(0.88, 1.82)	0.97	(0.80, 1.18)	1.40	(1.16, 1.70)	1.34	(1.10, 1.63)	1.15	(0.94, 1.41)
**Occupation**	**Non-physical labor**	1.00		1.00		1.00		1.00		1.00		1.00		1.00	
	**Physical labor**	1.21	(0.85, 1.70)	1.71	(1.09, 2.69)	1.14	(0.74, 1.76)	1.21	(0.95, 1.55)	1.07	(0.86, 1.33)	1.00	(0.82, 1.21)	1.37	(1.14, 1.65)
	**Unemployed**	1.07	(0.76, 1.49)	1.42	(0.93, 2.17)	1.25	(0.85, 1.83)	1.25	(1.01, 1.54)	1.14	(0.93, 1.39)	1.00	(0.83, 1.19)	1.41	(1.20, 1.66)
**Abdominal Obesity**															
**Income**	**1st Quartile**	1.00		1.00		1.00		1.00		1.00		1.00		1.00	
	**2nd Quartile**	1.10	(0.89, 1.37)	0.91	(0.67, 1.22)	0.77	(0.57, 1.05)	0.85	(0.71, 1.01)	0.83	(0.71, 0.97)	0.65	(0.55, 0.77)	0.87	(0.75, 1.00)
	**3rd Quartile**	0.88	(0.69, 1.12)	0.85	(0.65, 1.12)	1.07	(0.80, 1.44)	0.64	(0.55, 0.76)	0.78	(0.66, 0.92)	0.67	(0.57, 0.79)	0.69	(0.59, 0.81)
	**4th Quartile**	0.90	(0.71, 1.14)	0.89	(0.65, 1.21)	0.72	(0.51, 1.00)	0.69	(0.58, 0.83)	0.65	(0.54, 0.78)	0.54	(0.44, 0.65)	0.60	(0.51, 0.71)
**Education**	**High school or less**	1.00		1.00		1.00		1.00		1.00		1.00		1.00	
	**College or higher**	0.69	(0.48, 0.99)	0.48	(0.33, 0.69)	0.39	(0.25, 0.59)	0.53	(0.43, 0.65)	0.53	(0.43, 0.64)	0.58	(0.48, 0.70)	0.58	(0.50, 0.68)
**Region**	**Seoul**	1.00		1.00		1.00		1.00		1.00		1.00		1.00	
	**Metro urban**	0.80	(0.59, 1.07)	0.85	(0.64, 1.15)	1.26	(0.93, 1.70)	1.13	(0.89, 1.44)	1.60	(1.26, 2.03)	1.28	(1.04, 1.57)	0.81	(0.67, 0.99)
	**Non-metro urban**	0.86	(0.66, 1.13)	0.81	(0.59, 1.09)	1.10	(0.78, 1.54)	1.11	(0.87, 1.41)	1.43	(1.14, 1.79)	1.46	(1.20, 1.77)	0.94	(0.79, 1.11)
	**Rural**	0.81	(0.62, 1.05)	1.01	(0.71, 1.44)	0.99	(0.69, 1.42)	1.16	(0.91, 1.49)	1.74	(1.36, 2.22)	1.57	(1.25, 1.97)	1.05	(0.85, 1.29)
**Occupation**	**Non-physical labor**	1.00		1.00		1.00		1.00		1.00		1.00		1.00	
	**Physical labor**	1.41	(0.93, 2.15)	2.11	(1.23, 3.63)	1.19	(0.72, 1.98)	1.20	(0.94, 1.54)	0.99	(0.78, 1.26)	0.98	(0.77, 1.24)	1.35	(1.11, 1.65)
	**Unemployed**	1.39	(0.92, 2.09)	2.00	(1.20, 3.34)	1.19	(0.74, 1.92)	1.35	(1.07, 1.69)	1.08	(0.85, 1.37)	1.12	(0.91, 1.40)	1.58	(1.33, 1.88)

Note: Overall obesity was defined as BMI ≥ 25 kg/m^2^. Abdominal obesity was defined as waist circumference ≥85 cm in women. Mutually adjusted for age (19–29, 30–39, 40–49, 50–59, 60–69, 70–79 years), income level (1st, 2nd, 3rd, 4th quartiles), education level (some high school or less, some college or higher), region (Seoul, metro urban, non-metro urban, rural) and occupation (non-physical labor, physical labor, unemployed).

## Data Availability

The KNHANES data that support the findings of this study are available from “https://knhanes.cdc.go.kr/knhanes/main.do” (accessed on 25 February 2020).

## Supplementary Materials

The following are available online at https://www.mdpi.com/article/10.3390/ijerph18084162/s1, Figure S1: Participant flow chart; Figure S2: Age-standardized (A) mean BMI, (B) mean WC, and (C) mean WHtR among Korean men and women in the KNHANES I (1998)–VII (2016–2018); Figure S3: Trends of mean BMI and WC by birth cohort and age among Korean men and women in the KNHANES I (1998)–VII (2016–2018); Figure S4: Summary of obesity transition in Korea, Table S1: Total number of participants by sex and sociodemographic factors: the KNHANES I–VII; Table S2: Result of the segmented regression models predicting overall (BMI ≥ 25 kg/m^2^) and abdominal obesity prevalence by sex; Table S3: Mean BMI and WC by demographic and socioeconomic factors in men and women: the KNHANES I–VII; Table S4: The prevalence, odds ratios (ORs) and 95% confidence intervals (CIs) of overall and abdominal obesity by region and occupation type in women: the KNHANES I–VII; Table S5: Beta coefficients (β) and 95% confidence interval (CI) for the associations of socioeconomic factors (income, education, region, occupation) with mean BMI and WC in men: the KNHANES I–VII; Table S6: Beta coefficients (β) and 95% confidence interval (CI) for the associations of socioeconomic factors (income, education, region, occupation) with mean BMI and WC in women: the KNHANES I–VII.
