# Supplementary material for: Trends and Inequalities in Overall and Abdominal Obesity by Sociodemographic Factors in Korean Adults, 1998–2018
Source: Int J Environ Res Public Health. 2021 Apr 14;18(8):4162. doi: 10.3390/ijerph18084162 (PMC8070993; doi:10.3390/ijerph18084162)
Supplement: Supplementary file 1 [file ijerph-18-04162-s001.pdf]

**Trends and inequalities in overall and abdominal obesity by sociodemographic factors in Korean adults, 1998-2018, Jang et al.**

# SUPPLEMENTARY MATERIAL

## Figure S1. Participant flow chart

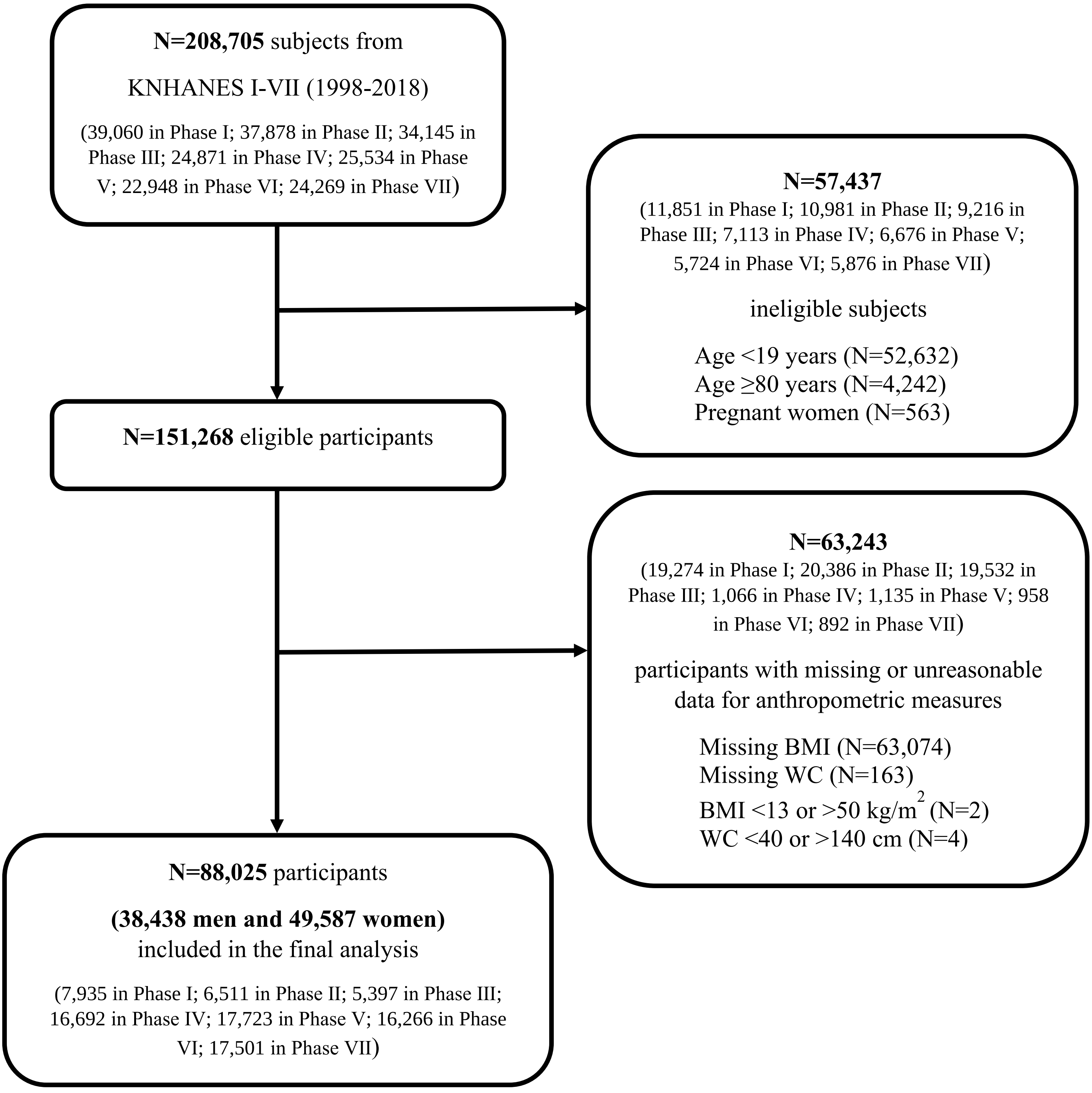

**Figure S2. Age-standardized (A) mean BMI, (B) mean WC, and (C) mean WHtR among Korean men and women in the KNHANES I (1998) - VII (2016-2018).** This figure shows the age-standardized mean values of (A) BMI, (B) WC, and (C) WHtR in Korean men and women from KNHANES I (1998) to VII (2016-2018). Age-standardization was performed using the 2005 Korean Census population as the reference population. The blue line indicates the age-standardized mean values in men. The red line indicates the age-standardized mean values in women. Beta and p-trend were estimated using weighted linear regression adjusted for age and survey design.

**(A) Mean BMI**

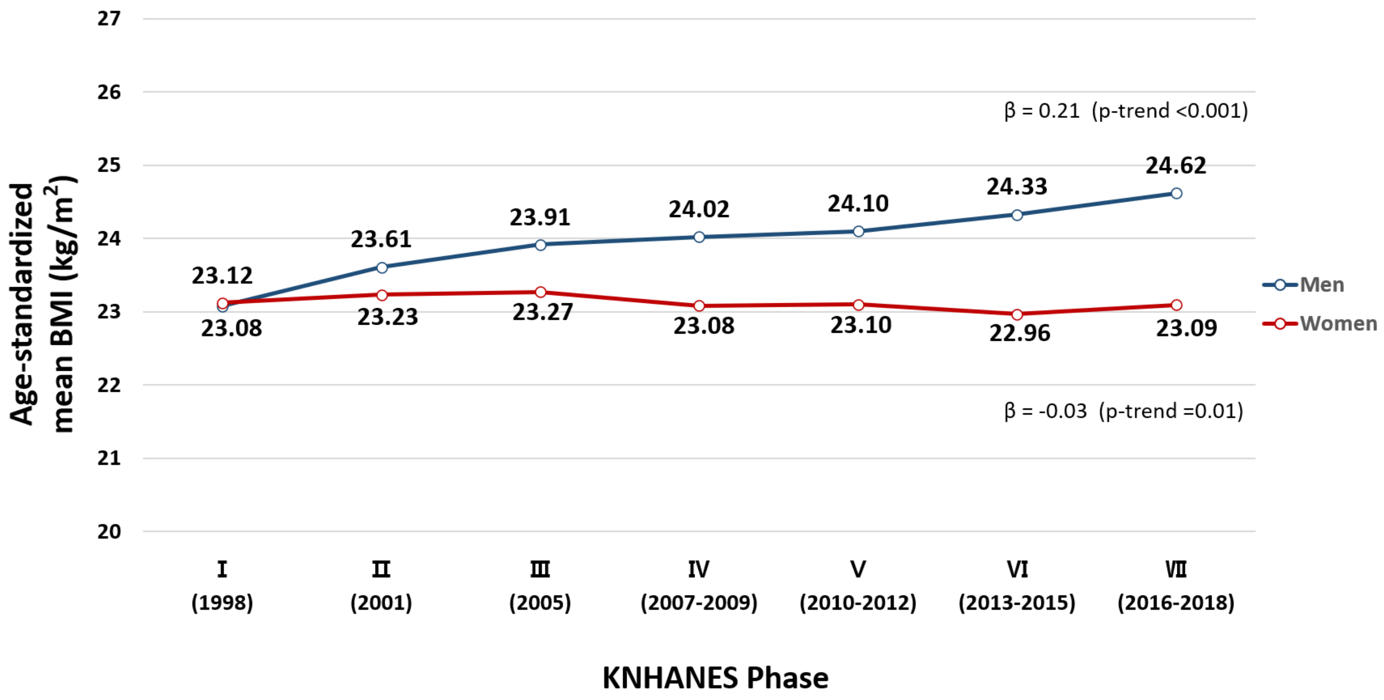


**(B) Mean WC**

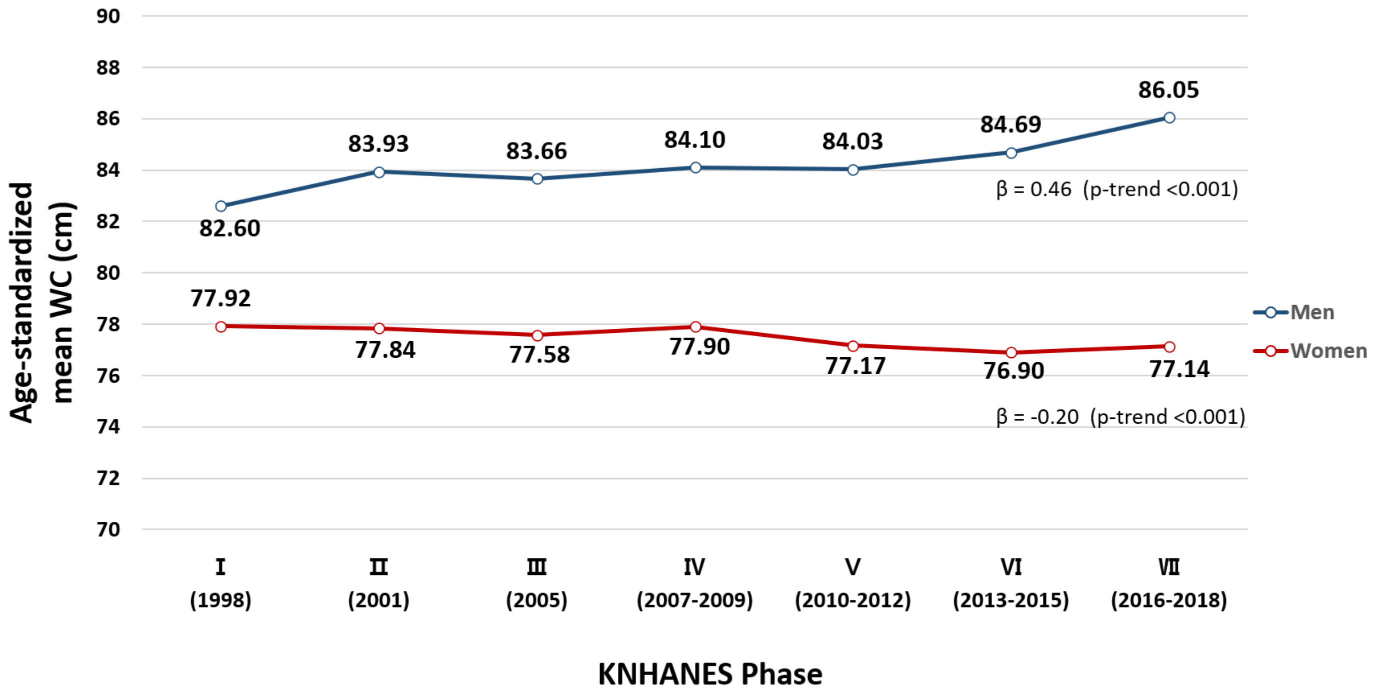

## (C) Mean WHtR

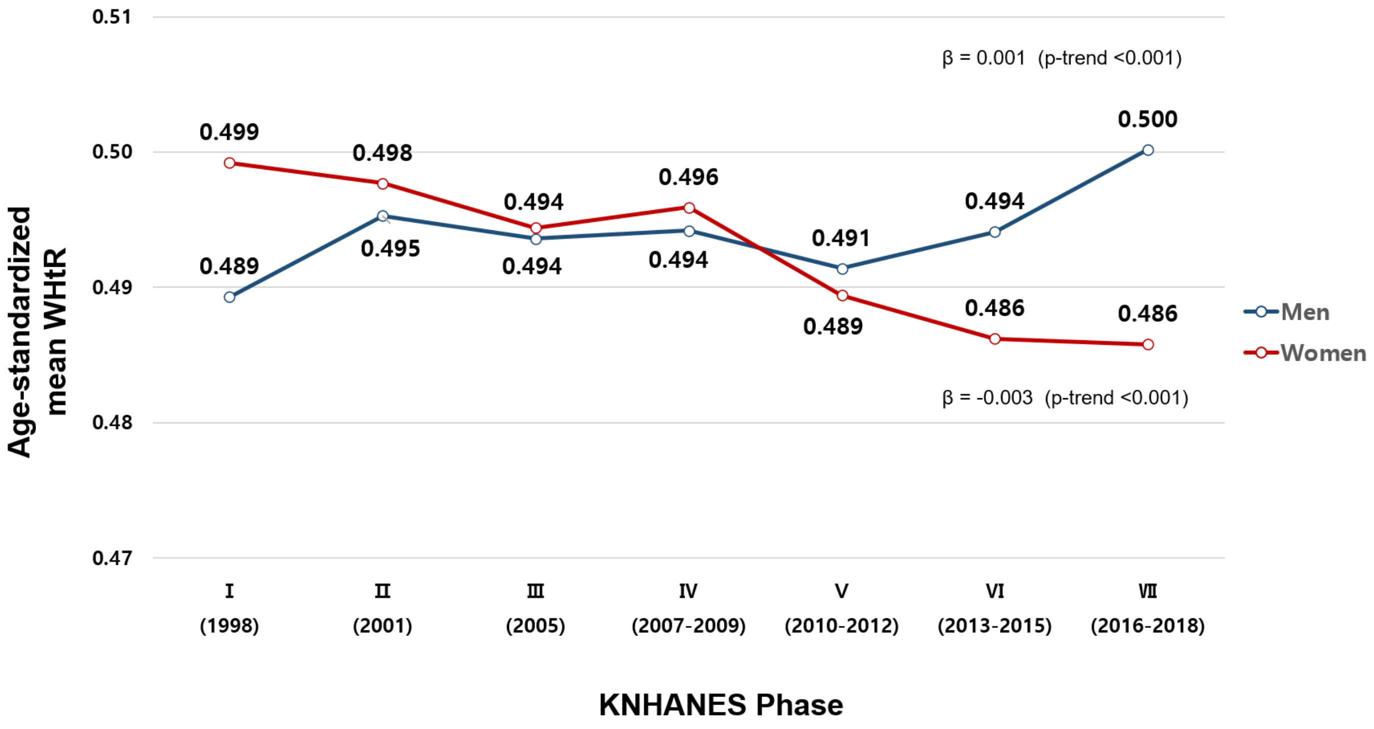

0.51
0.50
0.49
0.48
0.47
Age-standardized mean WHtR
β = 0.001 (p-trend <0.001)
β = -0.003 (p-trend <0.001)
0.499
0.498
0.494
0.496
0.489
0.486
0.486
0.489
0.495
0.494
0.494
0.491
0.494
0.500
Men
Women
I (1998)
II (2001)
III (2005)
IV (2007-2009)
V (2010-2012)
VI (2013-2015)
VII (2016-2018)
KNHANES Phase

**Figure S3. Trends of mean BMI and WC by birth cohort and age among Korean men and women in the KNHANES I (1998) - VII (2016-2018)**

This figure shows the trends in values of (A) mean BMI in Korean men, (B) mean WC in Korean men, (C) mean BMI in Korean women and (D) mean WC in Korean women by birth cohort and age using data from KNHANES I (1998) to VII (2016-2018).

**(A) Mean BMI in Korean men**

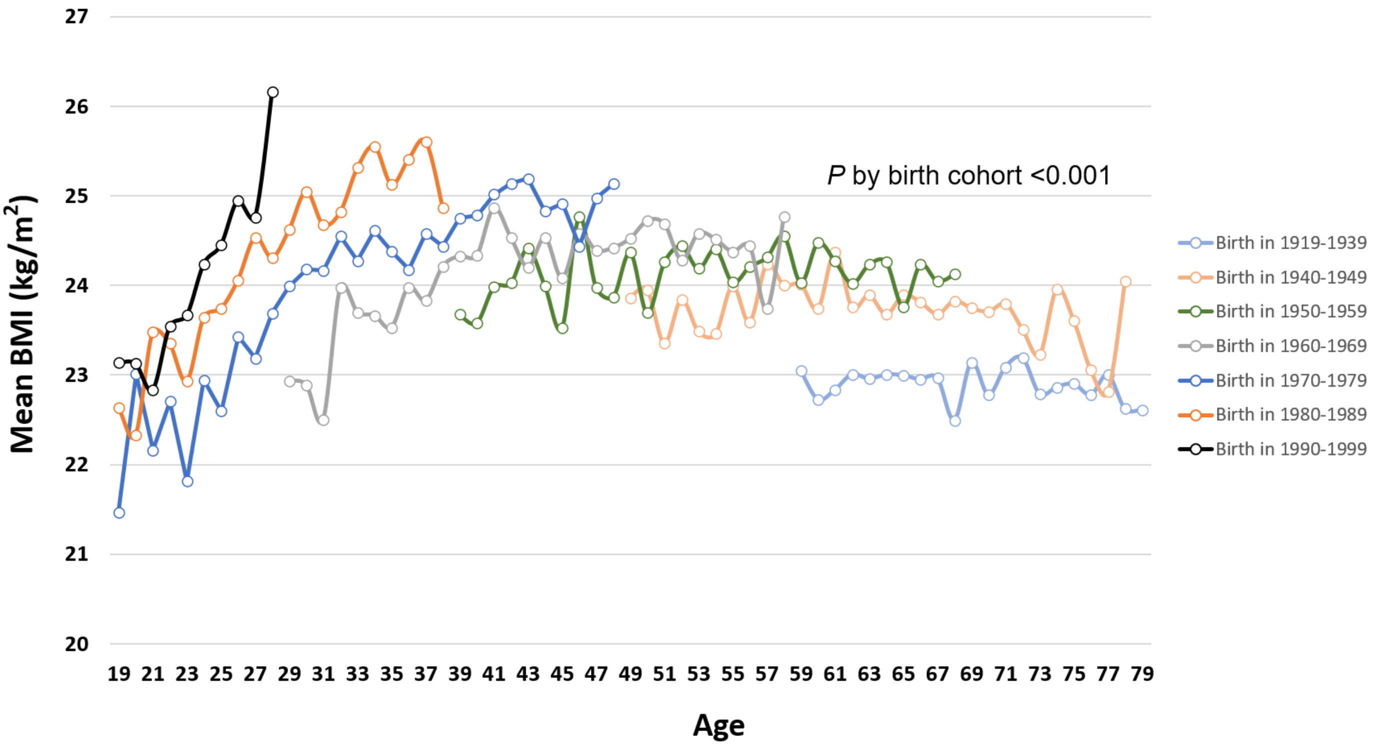


**(B) Mean WC in Korean men**

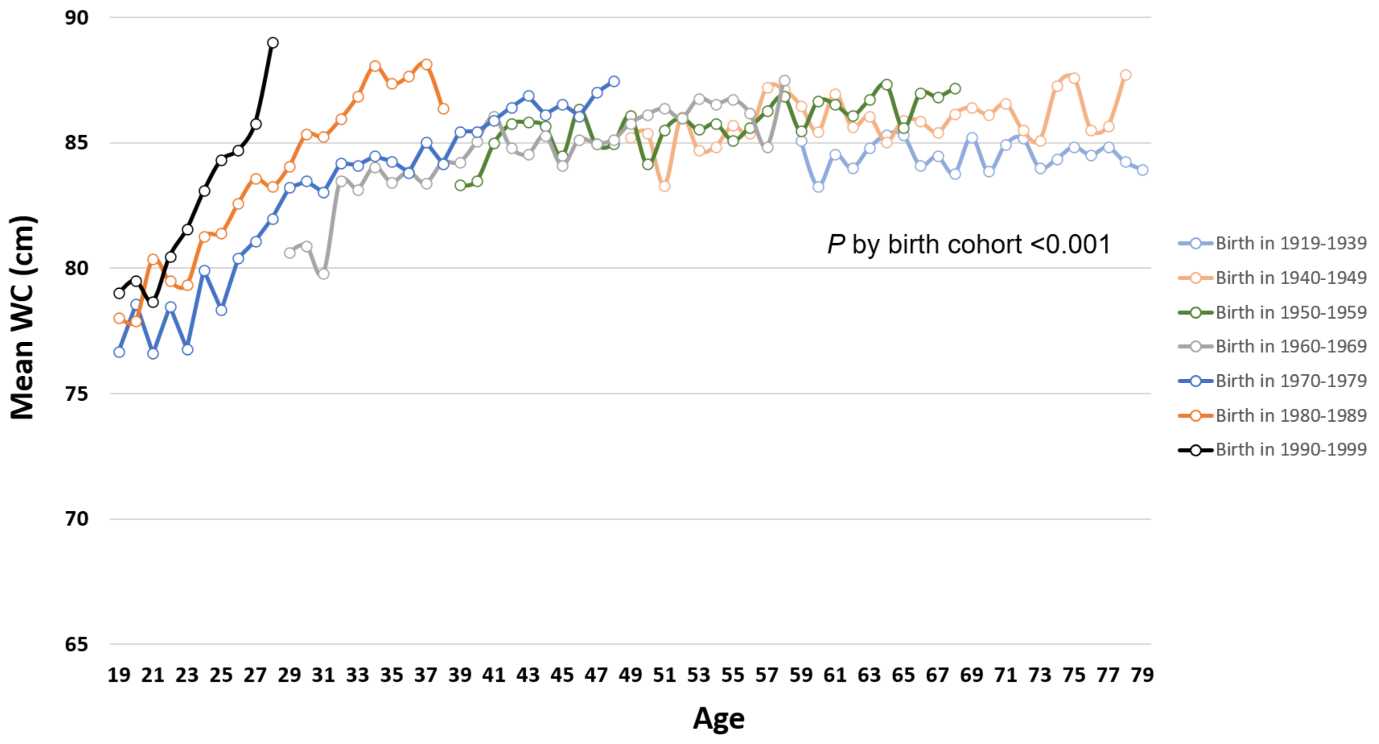

(C) Mean BMI in Korean women

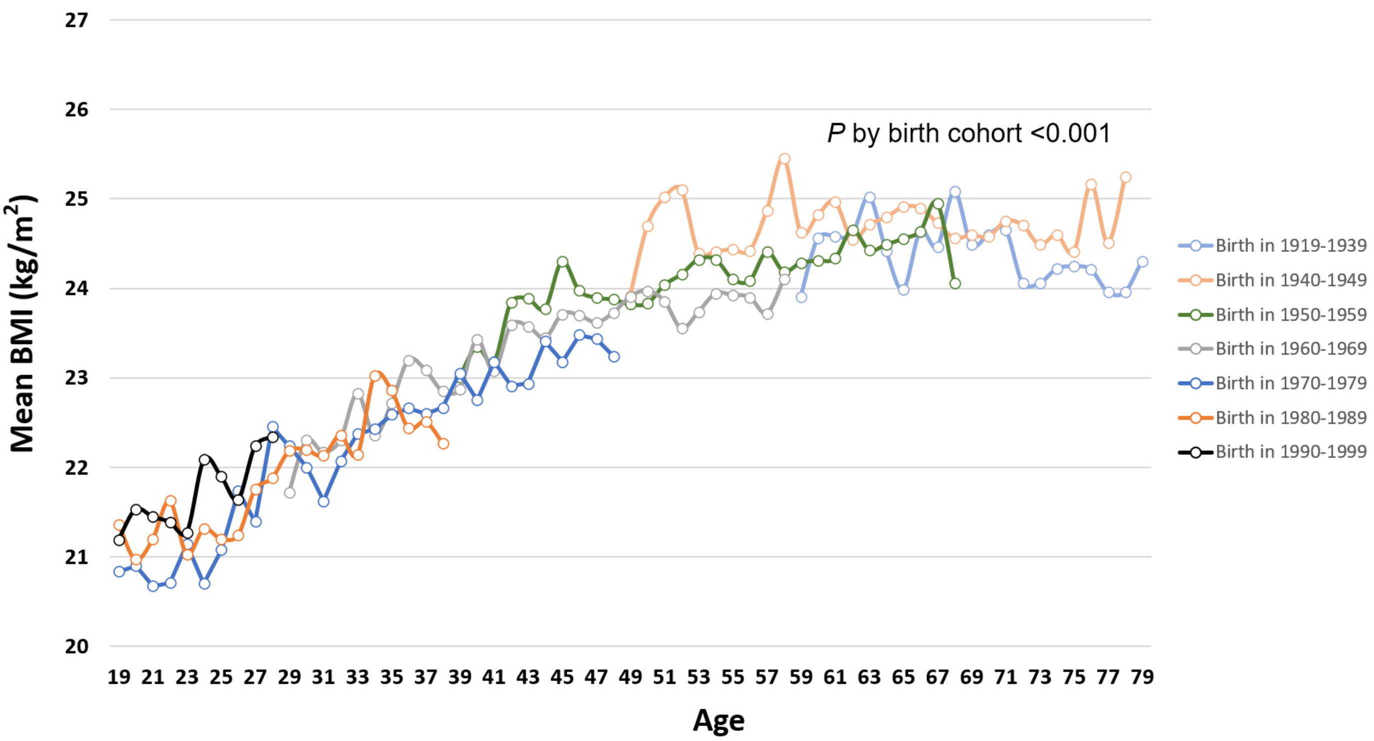


(D) Mean WC in Korean women

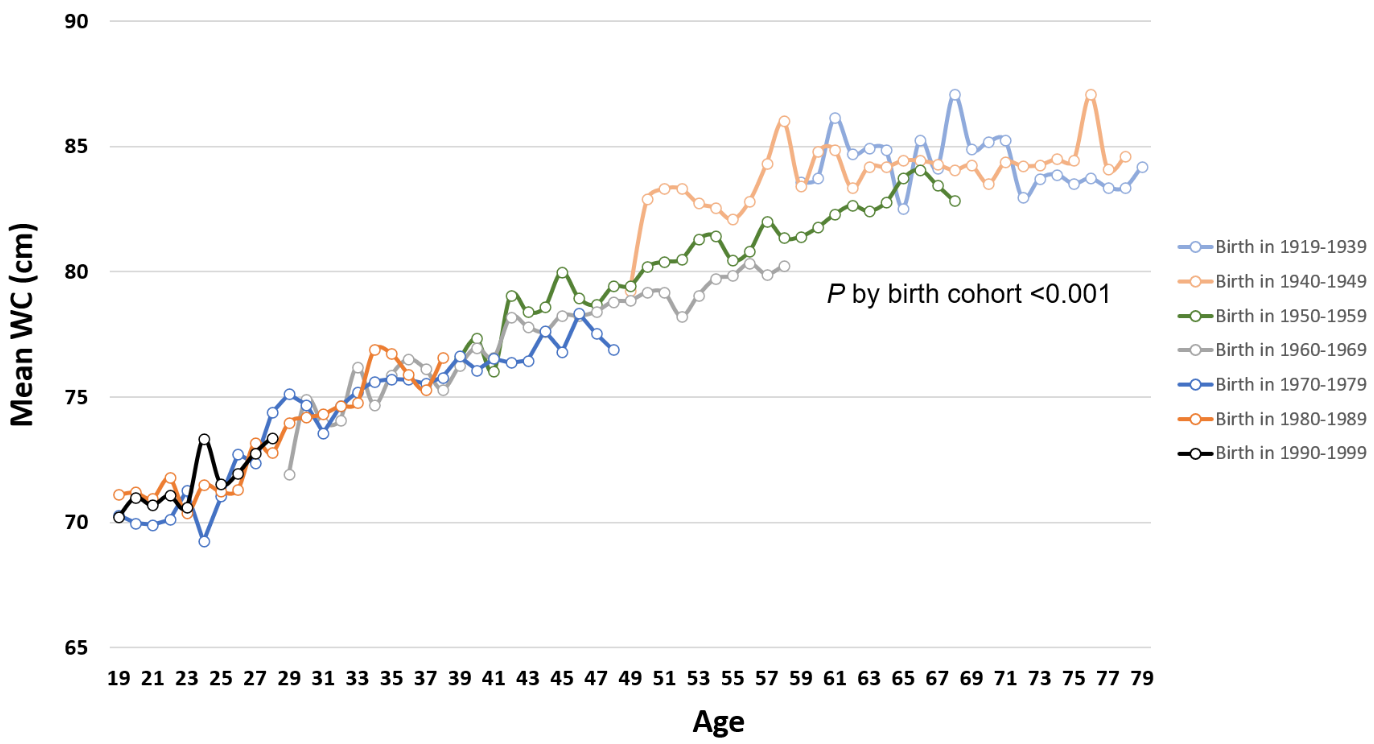

**Figure S4. Summary of obesity transition in Korea**

This figure summarizes the trends and inequalities in (A) overall obesity (BMI≥25kg/m$^2$) and (B) abdominal obesity prevalence by sex and socioeconomic status (SES) in Korean adults from KNHANES I (1998) to VII (2016-2018). The blue line is for men and the red line is for women. Brighter shade represents higher SES (the upper 50% of income levels and college or higher education) and darker shade represents lower SES (the lower 50% of income levels and high school or lower education). All results are age-standardized using the 2005 Korean Census population as the reference population.

**(A)**

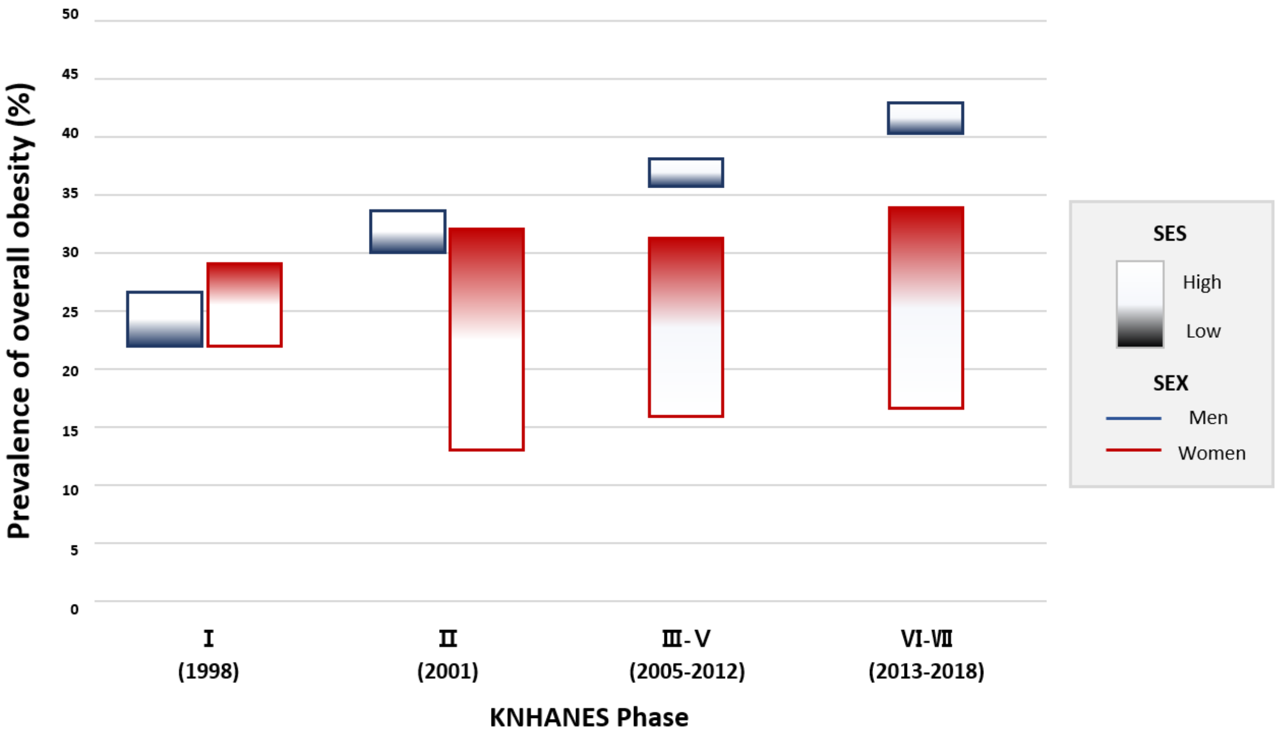


**(B)**

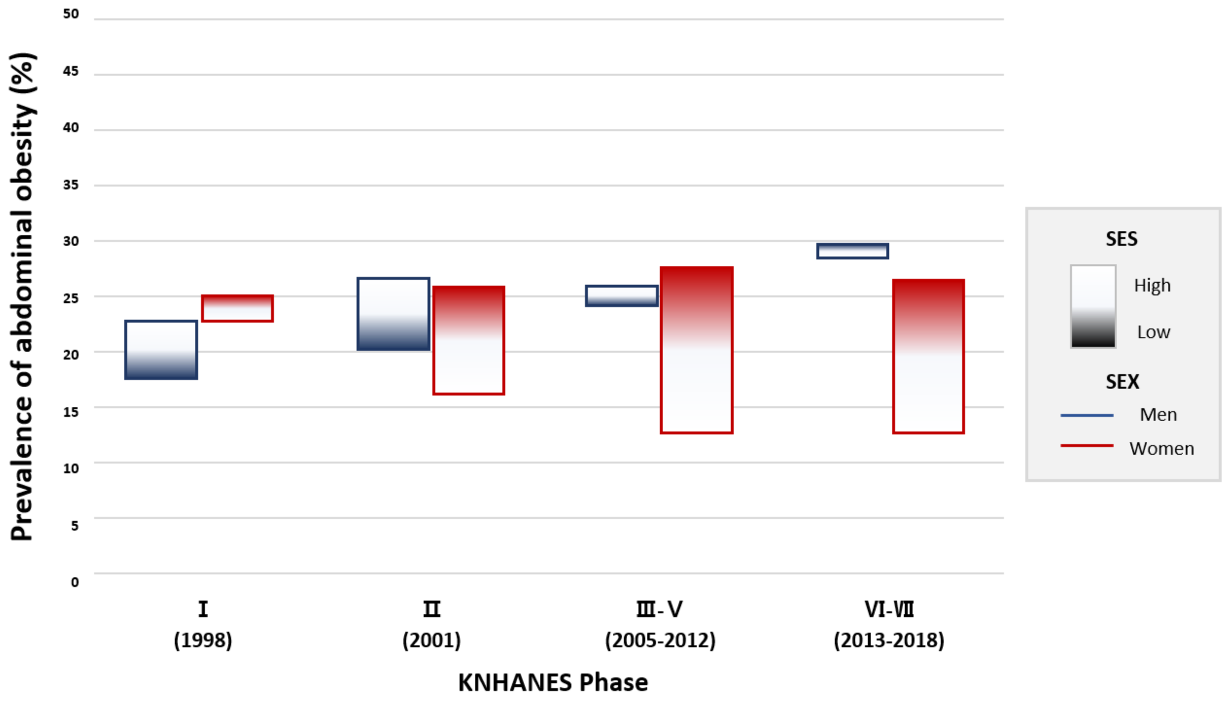


.

**Table S1. Total number of participants by sex and sociodemographic factors: the KNHANES I-VII**

| | | KNHANES | | | | | | | | | | | | | |
|---|---|---|---|---|---|---|---|---|---|---|---|---|---|---|---|
| | | Men | | | | | | | Women | | | | | | |
| Variables | | I (1998) | II (2001) | III (2005) | IV (07-09) | V (10-12) | VI (13-15) | VII (16-18) | I (1998) | II (2001) | III (2005) | IV (07-09) | V (10-12) | VI (13-15) | VII (16-18) |
| N | | 3634 | 2884 | 2327 | 7172 | 7609 | 7062 | 7750 | 4301 | 3627 | 3070 | 9520 | 10114 | 9204 | 9751 |
| | | N (%) | | | | | | | N (%) | | | | | | |
| Age | 19-29 years | 723 (28.6%) | 520 (26.8%) | 302 (23.1%) | 993 (21.6%) | 861 (20.3%) | 915 (20.0%) | 1007 (19.3%) | 883 (26.7%) | 659 (24.4%) | 422 (21.6%) | 1240 (19.7%) | 1225 (18.7%) | 1108 (17.8%) | 1153 (17.0%) |
| | 30-39 years | 923 (27.2%) | 727 (25.7%) | 496 (24.7%) | 1442 (23.3%) | 1378 (21.6%) | 1157 (20.1%) | 1283 (19.1%) | 1026 (25.5%) | 909 (24.2%) | 700 (22.8%) | 1969 (21.6%) | 1909 (20.0%) | 1525 (18.5%) | 1553 (17.2%) |
| | 40-49 years | 775 (19.8%) | 689 (22.0%) | 596 (23.5%) | 1475 (23.3%) | 1431 (22.7%) | 1329 (21.8%) | 1484 (21.2%) | 853 (19.0%) | 828 (21.3%) | 740 (22.6%) | 1922 (22.5%) | 1830 (21.9%) | 1760 (21.4%) | 1911 (21.0%) |
| | 50-59 years | 575 (13.0%) | 436 (13.1%) | 407 (14.5%) | 1243 (16.6%) | 1473 (18.7%) | 1403 (20.0%) | 1510 (20.5%) | 683 (13.2%) | 510 (13.1%) | 506 (14.5%) | 1652 (16.7%) | 2043 (18.7%) | 1927 (20.2%) | 2001 (20.8%) |
| | 60-69 years | 445 (8.2%) | 351 (8.9%) | 376 (9.4%) | 1199 (9.9%) | 1398 (10.3%) | 1317 (11.2%) | 1421 (12.9%) | 559 (10.2%) | 448 (10.8%) | 432 (10.9%) | 1574 (11.2%) | 1722 (11.4%) | 1584 (12.3%) | 1795 (14.0%) |
| | 70-79 years | 193 (3.2%) | 161 (3.6%) | 150 (4.7%) | 820 (5.4%) | 1068 (6.3%) | 941 (6.9%) | 1045 (7.0%) | 297 (5.6%) | 273 (6.2%) | 270 (7.7%) | 1163 (8.3%) | 1385 (9.3%) | 1300 (9.9%) | 1338 (10.0%) |
| Income | 1st Quartile | 843 (21.2%) | 628 (21.8%) | 604 (26.0%) | 1759 (24.5%) | 1815 (23.9%) | 1719 (24.3%) | 1897 (24.5%) | 1017 (23.6%) | 818 (22.6%) | 768 (25.0%) | 2303 (24.2%) | 2456 (24.3%) | 2250 (24.4%) | 2431 (24.9%) |
| | 2nd Quartile | 942 (25.7%) | 684 (23.7%) | 578 (24.8%) | 1723 (24.0%) | 1886 (24.8%) | 1775 (25.1%) | 1948 (25.1%) | 1113 (25.9%) | 812 (22.4%) | 789 (25.7%) | 2347 (24.7%) | 2536 (25.1%) | 2291 (24.9%) | 2441 (25.0%) |
| | 3rd Quartile | 959 (27.2%) | 718 (24.9%) | 547 (23.5%) | 1748 (24.4%) | 1904 (25.0%) | 1776 (25.1%) | 1940 (25.0%) | 1042 (24.2%) | 889 (24.5%) | 755 (24.6%) | 2315 (24.3%) | 2505 (24.8%) | 2317 (25.2%) | 2421 (24.8%) |
| | 4th Quartile | 890 (25.9%) | 694 (24.1%) | 575 (24.7%) | 1763 (24.6%) | 1902 (25.0%) | 1748 (24.8%) | 1941 (25.0%) | 1129 (26.2%) | 891 (24.6%) | 733 (23.9%) | 2300 (24.2%) | 2479 (24.5%) | 2289 (24.9%) | 2430 (24.9%) |
| | missing | 0 (0.0%) | 160 (5.5%) | 23 (1.0%) | 179 (2.5%) | 102 (1.3%) | 44 (0.6%) | 24 (0.3%) | 0 (0.0%) | 217 (6.0%) | 25 (0.8%) | 255 (2.7%) | 138 (1.4%) | 57 (0.6%) | 28 (0.3%) |
| Education | High school or less | 2622 (72.2%) | 1835 (63.6%) | 1424 (61.2%) | 4221 (58.9%) | 4098 (53.9%) | 3382 (47.9%) | 3529 (45.5%) | 3566 (82.9%) | 2779 (76.6%) | 2271 (74.0%) | 6859 (72.0%) | 6614 (65.4%) | 5434 (59.0%) | 5492 (56.3%) |
| | College or higher | 1012 (27.8%) | 1046 (36.3%) | 878 (37.7%) | 2872 (40.0%) | 3224 (42.4%) | 2940 (41.6%) | 3790 (48.9%) | 735 (17.1%) | 841 (23.2%) | 771 (25.1%) | 2588 (27.2%) | 3215 (31.8%) | 3054 (33.2%) | 3847 (39.5%) |
| | missing | 0 (0.0%) | 3 (0.1%) | 25 (1.1%) | 79 (1.1%) | 287 (3.8%) | 740 (10.5%) | 431 (5.6%) | 0 (0.0%) | 7 (0.2%) | 28 (0.9%) | 73 (0.8%) | 285 (2.8%) | 716 (7.8%) | 412 (4.2%) |
| Region | Seoul | 730 (20.1%) | 507 (17.6%) | 413 (17.7%) | 1182 (16.5%) | 1601 (21.0%) | 1340 (19.0%) | 1475 (19.0%) | 846 (19.7%) | 653 (18.0%) | 579 (18.9%) | 1623 (17.0%) | 2117 (20.9%) | 1845 (20.0%) | 1955 (20.0%) |
| | Metro urban | 771 (21.2%) | 845 (29.3%) | 602 (25.9%) | 1883 (26.3%) | 1930 (25.4%) | 1846 (26.1%) | 2158 (27.8%) | 923 (21.5%) | 1062 (29.3%) | 802 (26.1%) | 2534 (26.6%) | 2595 (25.7%) | 2463 (26.8%) | 2725 (27.9%) |
| | Non-metro urban | 920 (25.3%) | 892 (30.9%) | 778 (33.4%) | 2366 (33.0%) | 2600 (34.2%) | 2611 (37.0%) | 2893 (37.3%) | 1055 (24.5%) | 1125 (31.0%) | 1012 (33.0%) | 3134 (32.9%) | 3544 (35.0%) | 3382 (36.7%) | 3603 (37.0%) |

| | | | | | | | | | | | | | | | |
|---|---|---|---|---|---|---|---|---|---|---|---|---|---|---|---|
| | **Rural** | 1213 (33.4%) | 640 (22.2%) | 534 (22.9%) | 1741 (24.3%) | 1478 (19.4%) | 1265 (17.9%) | 1224 (15.8%) | 1477 (34.3%) | 787 (21.7%) | 677 (22.1%) | 2229 (23.4%) | 1858 (18.4%) | 1514 (16.4%) | 1468 (15.1%) |
| **Occupation** | **Non-physical labor** | 700 (19.3%) | 658 (22.8%) | 559 (24.0%) | 1706 (23.8%) | 2007 (26.4%) | 1724 (24.4%) | 2208 (28.5%) | 423 (9.8%) | 392 (10.8%) | 397 (12.9%) | 1348 (14.2%) | 1623 (16.0%) | 1623 (17.6%) | 2113 (21.7%) |
| | **Physical labor** | 2106 (58.0%) | 1526 (52.9%) | 1223 (52.6%) | 3571 (49.8%) | 3525 (46.3%) | 2886 (40.9%) | 3219 (41.5%) | 1643 (38.2%) | 1175 (32.4%) | 1059 (34.5%) | 3149 (33.1%) | 3144 (31.1%) | 2632 (28.6%) | 2915 (29.9%) |
| | **Unemployed** | 810 (22.3%) | 692 (24.0%) | 524 (22.5%) | 1763 (24.6%) | 1751 (23.0%) | 1691 (23.9%) | 1877 (24.2%) | 2235 (52.0%) | 2059 (56.8%) | 1614 (52.6%) | 4911 (51.6%) | 5062 (50.0%) | 4237 (46.0%) | 4313 (44.2%) |
| | **Missing** | 18 (0.5%) | 8 (0.3%) | 21 (0.9%) | 132 (1.8%) | 326 (4.3%) | 761 (10.8%) | 446 (5.8%) | 0 (0.0%) | 1 (0.0%) | 0 (0.0%) | 112 (1.2%) | 285 (2.8%) | 712 (7.7%) | 410 (4.2%) |
| **Birth cohort** | **Birth in 1919-1939** | 699 (18.9%) | 582 (9.1%) | 805 (10.8%) | 906 (11.5%) | 642 (8.8%) | 0 (0.0%) | 0 (0.0%) | 924 (18.1%) | 687 (8.8%) | 885 (9.3%) | 1025 (9.9%) | 780 (8.2%) | 0 (0.0%) | 0 (0.0%) |
| | **Birth in 1940-1949** | 435 (11.7%) | 428 (6.7%) | 622 (8.3%) | 753 (9.6%) | 527 (7.2%) | 119 (2.9%) | 0 (0.0%) | 612 (12.0%) | 529 (6.7%) | 711 (7.5%) | 967 (9.4%) | 636 (6.7%) | 172 (3.2%) | 0 (0.0%) |
| | **Birth in 1950-1959** | 283 (7.6%) | 419 (6.6%) | 478 (6.4%) | 542 (6.9%) | 428 (5.9%) | 177 (4.3%) | 0 (0.0%) | 431 (8.4%) | 460 (5.9%) | 588 (6.2%) | 744 (7.2%) | 591 (6.2%) | 256 (4.8%) | 0 (0.0%) |
| | **Birth in 1960-1969** | 912 (24.6%) | 1187 (18.5%) | 1278 (17.2%) | 1487 (19.0%) | 1371 (18.8%) | 891 (21.4%) | 46 (2.9%) | 1303 (25.5%) | 1555 (19.8%) | 1681 (17.7%) | 1952 (18.9%) | 1877 (19.7%) | 1108 (20.8%) | 44 (2.3%) |
| | **Birth in 1970-1979** | 797 (21.5%) | 1409 (22.0%) | 1459 (19.6%) | 1389 (17.7%) | 1479 (20.2%) | 911 (21.9%) | 165 (10.5%) | 1049 (20.5%) | 1724 (21.9%) | 1932 (20.4%) | 1866 (18.0%) | 1994 (20.9%) | 1258 (23.6%) | 291 (15.2%) |
| | **Birth in 1980-1989** | 424 (11.4%) | 1193 (18.6%) | 1379 (18.5%) | 1284 (16.4%) | 1327 (18.2%) | 943 (22.7%) | 512 (32.6%) | 558 (10.9%) | 1480 (18.8%) | 1787 (18.8%) | 1841 (17.8%) | 1752 (18.4%) | 1163 (21.8%) | 623 (32.4%) |
| | **Birth in 1990-1999** | 158 (4.3%) | 1187 (18.5%) | 1429 (19.2%) | 1480 (18.9%) | 1531 (20.9%) | 1115 (26.8%) | 850 (54.0%) | 234 (4.6%) | 1425 (18.1%) | 1912 (20.1%) | 1949 (18.8%) | 1892 (19.9%) | 1376 (25.8%) | 963 (50.1%) |

**Table S2. Result of the segmented regression models predicting overall (BMI≥25kg/m$^2$) and abdominal obesity prevalence by sex**

| | Men | | | | Women | | | |
|---|---|---|---|---|---|---|---|---|
| | Overall obesity | | | | | | | |
| | | 95% CI | | | | 95% CI | | |
| | β | Lower | Upper | P-value | β | Lower | Upper | P-value |
| **Intercept $\beta_0$** | 0.266 | 0.242 | 0.291 | <0.001 | 0.027 | 0.009 | 0.046 | 0.004 |
| **Baseline trend $\beta_1$** | 0.024 | 0.019 | 0.029 | <0.001 | -0.001 | -0.006 | 0.003 | 0.574 |
| **Prevalence change after phase VI $\beta_2$** | -0.023 | -0.055 | 0.008 | 0.146 | -0.025 | -0.050 | 0.001 | 0.063 |
| **Slope change after phase VI $\beta_3$** | 0.011 | -0.009 | 0.030 | 0.279 | 0.009 | -0.007 | 0.024 | 0.286 |
| | Abdominal obesity | | | | | | | |
| | | 95% CI | | | | 95% CI | | |
| | β | Lower | Upper | P-value | β | Lower | Upper | P-value |
| **Intercept $\beta_0$** | 0.098 | 0.077 | 0.119 | <0.001 | -0.081 | -0.099 | -0.062 | <0.001 |
| **Baseline trend $\beta_1$** | 0.010 | 0.005 | 0.015 | <0.001 | -0.003 | -0.008 | 0.001 | 0.142 |
| **Prevalence change after phase VI $\beta_2$** | -0.040 | -0.069 | -0.011 | 0.007 | -0.040 | -0.067 | -0.014 | 0.003 |
| **Slope change after phase VI $\beta_3$** | 0.041 | 0.023 | 0.059 | <0.001 | 0.014 | -0.002 | 0.030 | 0.085 |

The model used for the segmented regression was as follows. $Obesity\ prevalence\ at\ specific\ survey\ phase = \beta_0 + \beta_1 * Phase + \beta_2 * (PhaseVI\ or\ after) + \beta_3 * Time\ after\ PhaseVI + \beta_4 * Age + e_{Phase}$
where $\beta_0$ indicates intercept; $\beta_1$ indicates baseline trend by phases; $\beta_2$ indicates immediate prevalence change after Phase VI; $\beta_3$ indicates slope change after Phase VI; $\beta_4$ indicates age effect (10-year intervals, continuous); and $e_{Phase}$ indicates error term.

**Table S3. Mean BMI and WC by demographic and socioeconomic factors in men and women: the KNHANES I-VII**

| | | KNHANES | | | | | | | | | | | | | | | | | |
|---|---|---|---|---|---|---|---|---|---|---|---|---|---|---|---|---|---|---|---|
| **Mean BMI in men & women (kg/m²)** | | | | | | | | | | | | | | | | | | | |
| | | **Men** | | | | | | | | | **Women** | | | | | | | | |
| **Variables** | | **I (1998)** | **II (2001)** | **III (2005)** | **IV (2007-2009)** | **V (2010-2012)** | **VI (2013-2015)** | **VII (2016-2018)** | **Age-adjusted β** | **P for trend** | **I (1998)** | **II (2001)** | **III (2005)** | **IV (2007-2009)** | **V (2010-2012)** | **VI (2013-2015)** | **VII (2016-2018)** | **Age-adjusted β** | **P for trend** |
| **Age** | **19-29** | 22.5 | 23.0 | 23.2 | 23.7 | 23.7 | 23.7 | 24.3 | 0.24 | <0.001 | 21.3 | 21.2 | 21.6 | 21.5 | 21.4 | 21.5 | 21.9 | 0.07 | <0.05 |
| | **30-39** | 23.5 | 23.9 | 24.2 | 24.3 | 24.5 | 24.9 | 25.2 | 0.27 | <0.001 | 22.8 | 22.7 | 22.6 | 22.3 | 22.6 | 22.5 | 22.5 | -0.03 | 0.19 |
| | **40-49** | 23.9 | 24.2 | 24.5 | 24.3 | 24.5 | 24.8 | 24.9 | 0.15 | <0.001 | 23.7 | 24.0 | 23.8 | 23.5 | 23.6 | 23.2 | 23.4 | -0.10 | <0.001 |
| | **50-59** | 23.4 | 23.8 | 24.4 | 24.3 | 24.1 | 24.4 | 24.5 | 0.13 | <0.001 | 24.6 | 24.7 | 24.8 | 24.4 | 24.1 | 23.9 | 23.8 | -0.17 | <0.001 |
| | **60-69** | 22.6 | 23.2 | 23.7 | 23.7 | 23.9 | 24.0 | 24.2 | 0.21 | <0.001 | 24.3 | 24.9 | 24.8 | 24.9 | 24.7 | 24.5 | 24.5 | -0.05 | 0.06 |
| | **70-79** | 20.9 | 22.7 | 22.9 | 22.8 | 23.1 | 23.3 | 23.7 | 0.30 | <0.001 | 23.6 | 23.9 | 23.9 | 24.2 | 24.3 | 24.5 | 24.7 | 0.17 | <0.001 |
| **Income** | **1st Quartile** | 22.8 | 23.6 | 23.7 | 23.9 | 23.8 | 24.2 | 24.5 | 0.24 | <0.001 | 23.0 | 23.5 | 23.4 | 23.6 | 23.7 | 23.6 | 23.9 | 0.05 | 0.02 |
| | **2nd Quartile** | 23.2 | 23.4 | 23.8 | 24.0 | 24.2 | 24.4 | 24.5 | 0.22 | <0.001 | 23.2 | 23.4 | 23.5 | 23.4 | 23.4 | 23.3 | 23.5 | -0.05 | 0.02 |
| | **3rd Quartile** | 23.0 | 23.7 | 24.1 | 24.1 | 24.1 | 24.3 | 24.7 | 0.22 | <0.001 | 22.9 | 23.1 | 23.4 | 23.0 | 23.2 | 23.1 | 23.2 | -0.05 | 0.02 |
| | **4th Quartile** | 23.5 | 23.8 | 24.3 | 24.3 | 24.4 | 24.4 | 24.6 | 0.16 | <0.001 | 22.9 | 22.8 | 22.9 | 22.9 | 22.8 | 22.8 | 22.8 | -0.08 | <0.001 |
| **Education** | **High school or less** | 23.1 | 23.6 | 24.0 | 24.0 | 24.0 | 24.2 | 24.4 | 0.21 | <0.001 | 23.4 | 23.8 | 24.0 | 23.9 | 24.1 | 24.0 | 24.2 | 0.02 | 0.08 |
| | **College or higher** | 23.2 | 23.7 | 23.9 | 24.2 | 24.3 | 24.4 | 24.7 | 0.19 | <0.001 | 21.5 | 21.4 | 21.7 | 21.8 | 21.9 | 22.0 | 22.3 | 0.08 | <0.001 |
| **Region** | **Seoul** | 23.1 | 23.5 | 23.8 | 24.2 | 24.1 | 24.4 | 24.5 | 0.20 | <0.001 | 22.9 | 22.9 | 22.9 | 23.1 | 22.8 | 22.7 | 23.1 | -0.08 | <0.001 |
| | **Metro urban** | 23.3 | 23.7 | 24.1 | 23.9 | 24.1 | 24.2 | 24.5 | 0.16 | <0.001 | 22.8 | 23.0 | 23.3 | 23.2 | 23.2 | 23.2 | 23.2 | -0.04 | 0.06 |
| | **Non-metro urban** | 23.2 | 23.8 | 24.1 | 24.1 | 24.2 | 24.4 | 24.7 | 0.20 | <0.001 | 22.9 | 23.0 | 23.2 | 23.2 | 23.2 | 23.3 | 23.3 | -0.03 | 0.10 |
| | **Rural** | 22.8 | 23.4 | 23.7 | 24.1 | 24.0 | 24.4 | 24.7 | 0.28 | <0.001 | 23.4 | 23.9 | 23.9 | 23.3 | 24.1 | 23.8 | 24.1 | 0.06 | 0.02 |
| **Occupation** | **Non-physical labor** | 23.6 | 24.0 | 24.3 | 24.4 | 24.6 | 24.6 | 24.9 | 0.18 | <0.001 | 21.6 | 21.5 | 21.7 | 22.1 | 22.1 | 22.2 | 22.3 | 0.02 | 0.51 |
| | **Physical labor** | 23.2 | 23.7 | 24.1 | 24.0 | 24.0 | 24.3 | 24.6 | 0.19 | <0.001 | 23.5 | 23.9 | 23.9 | 23.6 | 23.8 | 23.6 | 23.8 | -0.03 | 0.09 |
| | **Unemployed** | 22.5 | 23.1 | 23.3 | 23.7 | 23.7 | 23.9 | 24.1 | 0.23 | <0.001 | 23.0 | 23.2 | 23.5 | 23.3 | 23.4 | 23.3 | 23.6 | -0.01 | 0.50 |
| **Mean WC in men & women (cm)** | | | | | | | | | | | | | | | | | | | |

| Variables | | Men | | | | | | | | | Women | | | | | | | | |
|---|---|---|---|---|---|---|---|---|---|---|---|---|---|---|---|---|---|---|---|
| | | I (1998) | II (2001) | III (2005) | IV (2007-2009) | V (2010-2012) | VI (2013-2015) | VII (2016-2018) | Age-adjusted β | P for trend | I (1998) | II (2001) | III (2005) | IV (2007-2009) | V (2010-2012) | VI (2013-2015) | VII (2016-2018) | Age-adjusted β | P for trend |
| **Age** | **19-29** | 79.0 | 79.9 | 79.5 | 81.5 | 81.1 | 81.4 | 83.3 | 0.62 | <0.001 | 71.7 | 71.1 | 71.9 | 72.7 | 71.1 | 71.7 | 71.9 | 0.004 | 0.95 |
| | **30-39** | 82.7 | 83.9 | 83.0 | 83.9 | 84.3 | 85.8 | 87.0 | 0.71 | <0.001 | 75.5 | 75.6 | 75.0 | 75.3 | 75.3 | 75.6 | 75.6 | 0.03 | 0.65 |
| | **40-49** | 85.1 | 86.1 | 85.2 | 85.0 | 84.9 | 85.5 | 86.6 | 0.20 | <0.001 | 78.9 | 78.6 | 78.1 | 78.1 | 78.0 | 76.9 | 77.7 | -0.25 | <0.001 |
| | **50-59** | 84.9 | 85.5 | 87.2 | 86.0 | 85.2 | 85.8 | 86.7 | 0.16 | 0.01 | 83.3 | 83.1 | 82.4 | 82.1 | 80.7 | 79.6 | 79.6 | -0.75 | <0.001 |
| | **60-69** | 83.5 | 85.1 | 85.3 | 85.9 | 85.8 | 85.8 | 87.1 | 0.43 | <0.001 | 84.5 | 85.1 | 84.4 | 85.1 | 83.8 | 82.8 | 82.9 | -0.45 | <0.001 |
| | **70-79** | 79.9 | 85.1 | 84.8 | 83.8 | 84.8 | 85.4 | 86.9 | 0.79 | <0.001 | 82.8 | 83.7 | 83.2 | 83.9 | 83.6 | 84.2 | 84.6 | 0.27 | <0.05 |
| **Income** | **1st Quartile** | 81.5 | 83.5 | 82.8 | 83.7 | 83.4 | 84.6 | 85.9 | 0.58 | <0.001 | 78.0 | 78.6 | 78.4 | 79.3 | 78.8 | 79.0 | 79.5 | -0.01 | 0.88 |
| | **2nd Quartile** | 82.4 | 82.9 | 83.2 | 84.0 | 84.4 | 84.8 | 86.0 | 0.52 | <0.001 | 77.8 | 78.3 | 77.9 | 79.0 | 78.2 | 77.7 | 78.7 | -0.22 | <0.001 |
| | **3rd Quartile** | 81.9 | 83.7 | 84.1 | 84.4 | 83.9 | 84.7 | 86.3 | 0.48 | <0.001 | 77.2 | 77.7 | 78.1 | 77.7 | 77.7 | 77.5 | 77.6 | -0.27 | <0.001 |
| | **4th Quartile** | 83.5 | 84.2 | 84.5 | 84.7 | 84.9 | 85.2 | 86.4 | 0.32 | <0.001 | 77.1 | 76.2 | 76.7 | 77.3 | 76.5 | 76.6 | 76.6 | -0.33 | <0.001 |
| **Education** | **High school or less** | 82.7 | 84.1 | 84.3 | 84.5 | 84.4 | 85.1 | 86.5 | 0.39 | <0.001 | 78.8 | 79.7 | 80.0 | 80.7 | 80.5 | 80.3 | 81.2 | -0.11 | <0.05 |
| | **College or higher** | 81.8 | 83.1 | 82.7 | 83.8 | 83.9 | 84.4 | 86.0 | 0.49 | <0.001 | 72.3 | 71.8 | 72.4 | 73.4 | 73.2 | 73.8 | 74.5 | 0.17 | <0.05 |
| **Region** | **Seoul** | 82.5 | 83.8 | 83.4 | 84.1 | 83.1 | 84.9 | 86.4 | 0.45 | <0.001 | 77.6 | 77.2 | 76.4 | 77.4 | 75.4 | 75.9 | 77.5 | -0.38 | <0.001 |
| | **Metro urban** | 82.8 | 83.7 | 84.1 | 84.0 | 85.0 | 84.7 | 85.6 | 0.31 | <0.001 | 76.5 | 76.9 | 78.3 | 78.8 | 78.4 | 77.9 | 77.6 | -0.25 | <0.001 |
| | **Non-metro urban** | 82.3 | 84.0 | 83.5 | 84.0 | 83.8 | 84.6 | 86.3 | 0.47 | <0.001 | 77.1 | 76.7 | 77.3 | 77.8 | 77.3 | 77.8 | 78.1 | -0.13 | 0.03 |
| | **Rural** | 81.9 | 83.0 | 83.4 | 84.9 | 84.7 | 85.2 | 86.6 | 0.65 | <0.001 | 79.3 | 80.8 | 79.7 | 80.0 | 81.0 | 79.8 | 80.4 | -0.02 | 0.81 |
| **Occupation** | **Non-physical labor** | 83.2 | 84.4 | 84.4 | 84.8 | 85.0 | 85.4 | 87.0 | 0.46 | <0.001 | 72.3 | 71.6 | 72.0 | 74.0 | 73.5 | 74.2 | 74.2 | 0.02 | 0.81 |
| | **Physical labor** | 83.0 | 84.1 | 83.8 | 84.1 | 84.0 | 84.6 | 86.0 | 0.34 | <0.001 | 79.2 | 79.2 | 79.2 | 79.4 | 79.4 | 78.7 | 79.6 | -0.23 | <0.001 |
| | **Unemployed** | 80.5 | 82.1 | 82.6 | 83.6 | 83.4 | 84.2 | 85.4 | 0.57 | <0.001 | 77.6 | 78.2 | 78.6 | 79.0 | 78.5 | 78.4 | 79.3 | -0.14 | <0.05 |

Values for mean BMI and WC are not adjusted for age. β coefficients were estimated from weighted linear regression models for mean BMI or WC values, adjusting for age (10-year intervals, continuous). Tests for trend were performed by including the KNHANES phase as a continuous variable in the models.

**Table S4. The prevalence, odds ratios (ORs) and 95% confidence intervals (CIs) of overall and abdominal obesity by region and occupation type in women: the KNHANES I-VII**

| | | Overall obesity | | | | Abdominal obesity | | | |
|---|---|---|---|---|---|---|---|---|---|
| | | Among unemployed and physical labor women | | Among non-physical labor women | | Among unemployed and physical labor women | | Among non-physical labor women | |
| **Phase** | | **Seoul and metro-urban** | **Rural and non-metro urban areas** | **Seoul and metro-urban** | **Rural and non-metro urban areas** | **Seoul and metro-urban** | **Rural and non-metro urban areas** | **Seoul and metro-urban** | **Rural and non-metro urban areas** |
| **I (1998)** | **Prevalence** | 26.0 % | 28.1 % | 13.7 % | 13.3 % | 22.1 % | 24.5 % | 7.9 % | 7.7 % |
| | **OR (95% CI)** | 1.0 (ref) | 0.97 (0.82, 1.16) | 1.0 (ref) | 0.94 (0.52, 1.70) | 1.0 (ref) | 0.94 (0.77, 1.15) | 1.0 (ref) | 1.00 (0.48, 2.09) |
| **II (2001)** | **Prevalence** | 27.3 % | 32.4 % | 9.0 % | 10.9 % | 23.9 % | 26.7 % | 5.1 % | 4.7 % |
| | **OR (95% CI)** | 1.0 (ref) | 1.15 (0.95, 1.38) | 1.0 (ref) | 1.29 (0.57, 2.93) | 1.0 (ref) | 0.98 (0.78, 1.23) | 1.0 (ref) | 0.92 (0.35, 2.42) |
| **III (2005)** | **Prevalence** | 29.3 % | 31.8 % | 10.9 % | 16.2 % | 24.9 % | 26.4 % | 7.8 % | 7.9 % |
| | **OR (95% CI)** | 1.0 (ref) | 1.02 (0.82, 1.26) | 1.0 (ref) | 1.59 (0.78, 3.25) | 1.0 (ref) | 0.92 (0.73, 1.15) | 1.0 (ref) | 1.00 (0.39, 2.53) |
| **IV (2007-2009)** | **Prevalence** | 29.3 % | 30.2 % | 14.5 % | 14.0 % | 26.7 % | 28.2 % | 9.7 % | 10.4 % |
| | **OR (95% CI)** | 1.0 (ref) | 1.04 (0.92, 1.17) | 1.0 (ref) | 1.00 (0.68, 1.48) | 1.0 (ref) | 1.03 (0.88, 1.21) | 1.0 (ref) | 1.15 (0.75, 1.77) |
| **V (2010-2012)** | **Prevalence** | 27.8 % | 33.4 % | 17.8 % | 17.6 % | 23.4 % | 28.9 % | 11.5 % | 13.0 % |
| | **OR (95% CI)** | 1.0 (ref) | **1.20 (1.05, 1.37)** | 1.0 (ref) | 0.88 (0.63, 1.21) | 1.0 (ref) | **1.18 (1.02, 1.36)** | 1.0 (ref) | 1.04 (0.70, 1.54) |
| **VI (2013-2015)** | **Prevalence** | 26.2 % | 31.4 % | 18.0 % | 17.2 % | 21.5 % | 27.2 % | 10.6 % | 12.0 % |
| | **OR (95% CI)** | 1.0 (ref) | **1.26 (1.12, 1.42)** | 1.0 (ref) | 0.90 (0.67, 1.22) | 1.0 (ref) | **1.31 (1.13, 1.51)** | 1.0 (ref) | 1.11 (0.76, 1.61) |
| **VII (2016-2018)** | **Prevalence** | 30.4 % | 33.1 % | 17.0 % | 16.7 % | 26.5 % | 29.2 % | 10.2 % | 12.0 % |
| | **OR (95% CI)** | 1.0 (ref) | 1.08 (0.96, 1.22) | 1.0 (ref) | 0.92 (0.70, 1.20) | 1.0 (ref) | 1.08 (0.94, 1.24) | 1.0 (ref) | 1.12 (0.83, 1.51) |

Prevalence is not age-adjusted.

ORs and CIs are adjusted for age (19-29, 30-39, 40-49, 50-59, 60-69, 70-79 years), income level (1st, 2nd, 3rd, 4th quartiles) and education level (some high school or less, some college or higher).

**Table S5. Beta coefficients (β) and 95% confidence intervals (CI) for the associations of socioeconomic factors (income, education, region, occupation) with mean BMI and WC in men: the KNHANES I-VII**

| Mean BMI in men | | | | | | | | | | | | | | | |
|---|---|---|---|---|---|---|---|---|---|---|---|---|---|---|---|
| | | KNHANES Phase (year) | | | | | | | | | | | | | |
| Variables | | I (1998) | | II (2001) | | III (2005) | | IV (2007-2009) | | V (2010-2012) | | VI (2013-2015) | | VII (2016-2018) | |
| | | β | (95% CI) | β | (95% CI) | β | (95% CI) | β | (95% CI) | β | (95% CI) | β | (95% CI) | β | (95% CI) |
| Income | 1st Quartile | 0.00 | | 0.00 | | 0.00 | | 0.00 | | 0.00 | | 0.00 | | 0.00 | |
| | 2nd Quartile | 0.33 | (0.00, 0.66) | -0.20 | (-0.58, 0.19) | 0.11 | (-0.37, 0.58) | 0.10 | (-0.18, 0.38) | 0.28 | (-0.03, 0.60) | 0.14 | (-0.16, 0.44) | -0.04 | (-0.33, 0.26) |
| | 3rd Quartile | 0.17 | (-0.18, 0.52) | 0.02 | (-0.35, 0.38) | 0.37 | (-0.17, 0.91) | 0.22 | (-0.09, 0.53) | 0.15 | (-0.17, 0.48) | -0.02 | (-0.29, 0.26) | 0.15 | (-0.14, 0.44) |
| | 4th Quartile | 0.50 | (0.13, 0.88) | 0.12 | (-0.29, 0.53) | 0.62 | (0.12, 1.12) | 0.34 | (0.05, 0.62) | 0.45 | (0.12, 0.78) | 0.17 | (-0.11, 0.46) | 0.07 | (-0.22, 0.36) |
| Education | High school or less | 0.00 | | 0.00 | | 0.00 | | 0.00 | | 0.00 | | 0.00 | | 0.00 | |
| | College or higher | 0.25 | (-0.04, 0.54) | 0.07 | (-0.27, 0.41) | -0.12 | (-0.56, 0.33) | 0.10 | (-0.15, 0.34) | 0.01 | (-0.26, 0.28) | 0.14 | (-0.11, 0.38) | 0.01 | (-0.24, 0.26) |
| Region | Seoul | 0.00 | | 0.00 | | 0.00 | | 0.00 | | 0.00 | | 0.00 | | 0.00 | |
| | Metro urban | 0.17 | (-0.19, 0.53) | 0.14 | (-0.27, 0.54) | 0.32 | (-0.19, 0.83) | -0.30 | (-0.59, -0.01) | 0.03 | (-0.26, 0.32) | -0.21 | (-0.49, 0.08) | 0.01 | (-0.27, 0.28) |
| | Non-metro urban | 0.08 | (-0.31, 0.46) | 0.18 | (-0.23, 0.58) | 0.19 | (-0.29, 0.66) | -0.12 | (-0.40, 0.16) | 0.16 | (-0.12, 0.44) | -0.07 | (-0.32, 0.19) | 0.23 | (-0.01, 0.48) |
| | Rural | 0.01 | (-0.39, 0.40) | -0.20 | (-0.61, 0.21) | -0.02 | (-0.55, 0.51) | 0.01 | (-0.33, 0.35) | 0.07 | (-0.28, 0.43) | 0.05 | (-0.29, 0.40) | 0.30 | (-0.06, 0.65) |
| Occupation | Non-physical labor | 0.00 | | 0.00 | | 0.00 | | 0.00 | | 0.00 | | 0.00 | | 0.00 | |
| | Physical labor | -0.09 | (-0.37, 0.19) | -0.17 | (-0.56, 0.21) | -0.05 | (-0.49, 0.39) | -0.18 | (-0.44, 0.08) | -0.43 | (-0.70, -0.16) | -0.04 | (-0.29, 0.20) | -0.23 | (-0.47, 0.01) |
| | Unemployed | -0.30 | (-0.69, 0.10) | -0.34 | (-0.82, 0.15) | -0.32 | (-0.89, 0.25) | -0.13 | (-0.47, 0.21) | -0.45 | (-0.81, -0.09) | 0.05 | (-0.27, 0.38) | -0.36 | (-0.69, -0.03) |

| Mean WC in men | | | | | | | | | | | | | | | |
|---|---|---|---|---|---|---|---|---|---|---|---|---|---|---|---|
| | | KNHANES Phase (year) | | | | | | | | | | | | | |
| Variables | | I (1998) | | II (2001) | | III (2005) | | IV (2007-2009) | | V (2010-2012) | | VI (2013-2015) | | VII (2016-2018) | |
| | | β | (95% CI) | β | (95% CI) | β | (95% CI) | β | (95% CI) | β | (95% CI) | β | (95% CI) | β | (95% CI) |
| Income | 1st Quartile | 0.00 | | 0.00 | | 0.00 | | 0.00 | | 0.00 | | 0.00 | | 0.00 | |
| | 2nd Quartile | 0.74 | (-0.16, 1.64) | -0.53 | (-1.54, 0.48) | 0.32 | (-0.95, 1.60) | 0.26 | (-0.53, 1.05) | 0.74 | (-0.11, 1.59) | 0.25 | (-0.53, 1.04) | -0.15 | (-0.93, 0.62) |

| | | | | | | | | | | | | | | | |
|---|---|---|---|---|---|---|---|---|---|---|---|---|---|---|---|
| | **3rd Quartile** | 0.23 | (-0.71, 1.16) | -0.12 | (-1.16, 0.92) | 0.80 | (-0.62, 2.22) | 0.67 | (-0.14, 1.49) | 0.14 | (-0.73, 1.01) | -0.20 | (-0.98, 0.58) | 0.13 | (-0.63, 0.88) |
| | **4th Quartile** | 1.31 | (0.38, 2.25) | 0.43 | (-0.66, 1.53) | 1.49 | (0.15, 2.84) | 0.79 | (0.03, 1.56) | 1.03 | (0.13, 1.93) | 0.31 | (-0.49, 1.11) | 0.02 | (-0.74, 0.78) |
| **Education** | **High school or less** | 0.00 | | 0.00 | | 0.00 | | 0.00 | | 0.00 | | 0.00 | | 0.00 | |
| | **College or higher** | 0.39 | (-0.40, 1.17) | 0.15 | (-0.71, 1.02) | 0.05 | (-1.14, 1.25) | 0.33 | (-0.33, 0.99) | 0.37 | (-0.36, 1.10) | 0.35 | (-0.30, 1.01) | -0.10 | (-0.75, 0.54) |
| **Region** | **Seoul** | 0.00 | | 0.00 | | 0.00 | | 0.00 | | 0.00 | | 0.00 | | 0.00 | |
| | **Metro urban** | 0.24 | (-0.84, 1.33) | -0.12 | (-1.19, 0.96) | 0.72 | (-0.60, 2.05) | -0.11 | (-1.00, 0.77) | 2.01 | (1.17, 2.84) | -0.11 | (-0.87, 0.66) | -0.73 | (-1.51, 0.05) |
| | **Non-metro urban** | -0.30 | (-1.47, 0.86) | -0.06 | (-1.09, 0.97) | -0.01 | (-1.28, 1.25) | -0.28 | (-1.16, 0.60) | 0.97 | (0.11, 1.83) | -0.13 | (-0.88, 0.62) | -0.06 | (-0.76, 0.65) |
| | **Rural** | -0.61 | (-1.79, 0.58) | -1.43 | (-2.58, -0.28) | -0.60 | (-2.02, 0.83) | 0.55 | (-0.40, 1.50) | 1.70 | (0.66, 2.73) | 0.20 | (-0.81, 1.20) | 0.20 | (-0.75, 1.15) |
| **Occupation** | **Non-physical labor** | 0.00 | | 0.00 | | 0.00 | | 0.00 | | 0.00 | | 0.00 | | 0.00 | |
| | **Physical labor** | -0.28 | (-1.10, 0.53) | -0.43 | (-1.40, 0.53) | -0.78 | (-1.99, 0.42) | -0.70 | (-1.39, -0.01) | -1.24 | (-1.95, -0.52) | -0.74 | (-1.43, -0.06) | -1.06 | (-1.69, -0.42) |
| | **Unemployed** | -0.86 | (-1.97, 0.26) | -0.84 | (-2.01, 0.34) | -0.91 | (-2.53, 0.71) | -0.49 | (-1.38, 0.40) | -0.97 | (-1.92, -0.01) | 0.01 | (-0.86, 0.87) | -0.91 | (-1.76, -0.06) |

Mutually adjusted for age (19-29, 30-39, 40-49, 50-59, 60-69, 70-79 years), income level (1$^{st}$, 2$^{nd}$, 3$^{rd}$, 4$^{th}$ quartiles), education level (high school or less, college or higher), region (Seoul, metro urban, non-metro urban, rural) and occupation (non-physical labor, physical labor, unemployed).

**Table S6. Beta coefficients (β) and 95% confidence intervals (CI) for the associations of socioeconomic factors (income, education, region, occupation) with mean BMI and WC in women: the KNHANES I-VII**

**Mean BMI in women**

| Variables | | I (1998) | | II (2001) | | III (2005) | | IV (2007-2009) | | V (2010-2012) | | VI (2013-2015) | | VII (2016-2018) | |
|---|---|---|---|---|---|---|---|---|---|---|---|---|---|---|---|
| | | β | (95% CI) | β | (95% CI) | β | (95% CI) | β | (95% CI) | β | (95% CI) | β | (95% CI) | β | (95% CI) |
| **Income** | **1st Quartile** | 0.00 | | 0.00 | | 0.00 | | 0.00 | | 0.00 | | 0.00 | | 0.00 | |
| | **2nd Quartile** | 0.15 | (-0.16, 0.46) | -0.01 | (-0.40, 0.39) | 0.18 | (-0.20, 0.56) | -0.16 | (-0.43, 0.10) | -0.23 | (-0.50, 0.03) | -0.29 | (-0.53, -0.04) | -0.34 | (-0.58, -0.11) |
| | **3rd Quartile** | -0.04 | (-0.35, 0.28) | -0.19 | (-0.57, 0.19) | 0.26 | (-0.15, 0.67) | -0.46 | (-0.72, -0.20) | -0.28 | (-0.53, -0.03) | -0.41 | (-0.67, -0.15) | -0.50 | (-0.76, -0.25) |
| | **4th Quartile** | -0.02 | (-0.32, 0.29) | -0.28 | (-0.70, 0.14) | -0.14 | (-0.55, 0.28) | -0.57 | (-0.83, -0.31) | -0.51 | (-0.78, -0.24) | -0.63 | (-0.89, -0.36) | -0.69 | (-0.94, -0.45) |
| **Education** | **High school or less** | 0.00 | | 0.00 | | 0.00 | | 0.00 | | 0.00 | | 0.00 | | 0.00 | |
| | **College or higher** | -0.78 | (-1.08, -0.47) | -1.06 | (-1.39, -0.72) | -1.16 | (-1.53, -0.79) | -1.08 | (-1.34, -0.81) | -1.13 | (-1.38, -0.87) | -0.87 | (-1.10, -0.64) | -0.99 | (-1.22, -0.76) |
| **Region** | **Seoul** | 0.00 | | 0.00 | | 0.00 | | 0.00 | | 0.00 | | 0.00 | | 0.00 | |
| | **Metro urban** | -0.06 | (-0.35, 0.23) | -0.09 | (-0.45, 0.27) | 0.14 | (-0.27, 0.55) | -0.03 | (-0.28, 0.22) | 0.31 | (0.02, 0.60) | 0.28 | (0.01, 0.54) | -0.05 | (-0.31, 0.21) |
| | **Non-metro urban** | -0.06 | (-0.32, 0.21) | -0.05 | (-0.42, 0.32) | 0.16 | (-0.25, 0.58) | 0.14 | (-0.12, 0.40) | 0.23 | (-0.20, 0.48) | 0.46 | (0.22, 0.70) | 0.03 | (-0.19, 0.26) |
| | **Rural** | -0.07 | (-0.38, 0.23) | 0.13 | (-0.30, 0.56) | 0.34 | (-0.14, 0.82) | -0.18 | (-0.44, 0.08) | 0.62 | (0.32, 0.93) | 0.54 | (0.25, 0.83) | 0.31 | (-0.04, 0.66) |
| **Occupation** | **Non-physical labor** | 0.00 | | 0.00 | | 0.00 | | 0.00 | | 0.00 | | 0.00 | | 0.00 | |
| | **Physical labor** | 0.27 | (-0.09, 0.64) | 0.46 | (0.02, 0.89) | 0.45 | (-0.07, 0.97) | -0.09 | (-0.40, 0.23) | 0.10 | (-0.20, 0.39) | 0.05 | (-0.22, 0.31) | 0.41 | (0.14, 0.68) |
| | **Unemployed** | 0.23 | (-0.08, 0.55) | 0.14 | (-0.23, 0.50) | 0.55 | (0.10, 0.99) | 0.11 | (-0.15, 0.37) | 0.15 | (-0.12, 0.42) | -0.01 | (-0.26, 0.23) | 0.40 | (0.16, 0.63) |

**Mean WC in women**

| Variables | | I (1998) | | II (2001) | | III (2005) | | IV (2007-2009) | | V (2010-2012) | | VI (2013-2015) | | VII (2016-2018) | |
|---|---|---|---|---|---|---|---|---|---|---|---|---|---|---|---|
| | | β | (95% CI) | β | (95% CI) | β | (95% CI) | β | (95% CI) | β | (95% CI) | β | (95% CI) | β | (95% CI) |
| **Income** | **1st Quartile** | 0.00 | | 0.00 | | 0.00 | | 0.00 | | 0.00 | | 0.00 | | 0.00 | |
| | **2nd Quartile** | 0.16 | (-0.61, 0.94) | 0.10 | (-0.92, 1.12) | -0.05 | (-1.07, 0.98) | -0.50 | (-1.19, 0.19) | -0.66 | (-1.30, -0.01) | -1.14 | (-1.80, -0.48) | -0.67 | (-1.29, -0.05) |

| | | | | | | | | | | | | | | | |
|---|---|---|---|---|---|---|---|---|---|---|---|---|---|---|---|
| | **3rd Quartile** | -0.40 | (-1.20, 0.41) | -0.27 | (-1.21, 0.67) | 0.23 | (-0.88, 1.34) | -1.29 | (-1.97, -0.61) | -0.70 | (-1.33, -0.06) | -1.20 | (-1.90, -0.49) | -1.39 | (-2.03, -0.74) |
| | **4th Quartile** | -0.60 | (-1.44, 0.24) | -0.93 | (-2.02, 0.16) | -0.76 | (-1.89, 0.37) | -1.67 | (-2.40, -0.95) | -1.49 | (-2.15, -0.83) | -2.03 | (-2.74, -1.33) | -2.14 | (-2.77, -1.51) |
| **Education** | **High school or less** | 0.00 | | 0.00 | | 0.00 | | 0.00 | | 0.00 | | 0.00 | | 0.00 | |
| | **College or higher** | -1.89 | (-2.77, -1.02) | -3.07 | (-3.96, -2.19) | -3.07 | (-3.97, -2.17) | -3.10 | (-3.78, -2.41) | -2.62 | (-3.24, -2.00) | -2.41 | (-3.02, -1.81) | -2.37 | (-2.94, -1.80) |
| **Region** | **Seoul** | 0.00 | | 0.00 | | 0.00 | | 0.00 | | 0.00 | | 0.00 | | 0.00 | |
| | **Metro urban** | -1.14 | (-2.14, -0.14) | -0.93 | (-1.98, 0.12) | 1.13 | (0.07, 2.19) | 0.90 | (-0.09, 1.89) | 2.72 | (1.88, 3.56) | 1.45 | (0.65, 2.26) | -0.56 | (-1.32, 0.20) |
| | **Non-metro urban** | -0.78 | (-1.72, 0.15) | -0.89 | (-1.90, 0.12) | 0.54 | (-0.59, 1.67) | 0.42 | (-0.62, 1.45) | 1.65 | (0.86, 2.45) | 1.66 | (0.93, 2.39) | 0.07 | (-0.62, 0.77) |
| | **Rural** | -1.22 | (-2.19, -0.24) | 0.27 | (-0.93, 1.47) | 0.64 | (-0.70, 1.98) | 0.86 | (-0.26, 1.99) | 3.27 | (2.39, 4.14) | 1.99 | (1.09, 2.90) | 0.65 | (-0.32, 1.62) |
| **Occupation** | **Non-physical labor** | 0.00 | | 0.00 | | 0.00 | | 0.00 | | 0.00 | | 0.00 | | 0.00 | |
| | **Physical labor** | 1.26 | (0.18, 2.34) | 1.28 | (0.05, 2.51) | 1.26 | (-0.10, 2.63) | -0.15 | (-0.91, 0.61) | 0.60 | (-0.13, 1.32) | -0.05 | (-0.76, 0.65) | 1.25 | (0.61, 1.88) |
| | **Unemployed** | 1.02 | (0.13, 1.92) | 1.01 | (-0.02, 2.03) | 1.82 | (0.64, 3.00) | 0.66 | (0.00, 1.33) | 0.94 | (0.28, 1.60) | 0.17 | (-0.44, 0.79) | 1.53 | (0.96, 2.09) |

Mutually adjusted for age (19-29, 30-39, 40-49, 50-59, 60-69, 70-79 years), income level (1st, 2nd, 3rd, 4th quartiles), education level (high school or less, college or higher), region (Seoul, metro urban, non-metro urban, rural) and occupation (non-physical labor, physical labor, unemployed).
